# Vojta therapy improves postural control in very early stroke rehabilitation: a randomised controlled pilot trial

**DOI:** 10.1186/s42466-020-00070-4

**Published:** 2020-08-20

**Authors:** Corina Epple, Barbara Maurer-Burkhard, Mari-Carmen Lichti, Thorsten Steiner

**Affiliations:** 1 Department of Neurology, Klinikum Frankfurt Höchst, Frankfurt, Germany; 2 Department of Neurology, Klinikum Hanau, Hanau, Germany; 3 Department of Neurology, Heidelberg University Hospital, Heidelberg, Germany

**Keywords:** Vojta therapy, Stroke recovery, Postural control, Rehabilitation, Acute stroke, Physiotherapy

## Abstract

**Background:**

It is still unclear, which physiotherapeutic approaches are most effective in stroke recovery. Vojta therapy is a type of physiotherapy that was originally developed for children and adolescents with cerebral palsy. Vojta therapy has been reported to improve automatic control of body posture. Because acute stroke patients are characterised by a disturbance in the ability to adapt to changes in body position, requiring automatic postural adjustment, we decided to investigate Vojta therapy in the early rehabilitation of stroke patients. Aim of the trial was to test the hypothesis that Vojta therapy - as a new physiotherapeutic approach in early stroke recovery - improves postural control and motor function in patients with acute ischaemic stroke (AIS) or intracerebral haemorrhage (ICH).

**Methods:**

This prospective, randomised controlled trial included patients with imaging-confirmed AIS or ICH, severe hemiparesis and randomly assigned them to Vojta therapy or standard physiotherapy within 72 h after stroke onset. Main exclusion criterion was restricted ability to communicate. Primary endpoint was the improvement of postural control measured by the Trunk Control Test (TCT) on day 9 after admission. Secondary endpoint among others was improvement of arm function (measured with Motor Evaluation Scale for Upper Extremity in Stroke Patients [MESUPES]).

**Results:**

Forty patients (20 per group) were randomised into the trial. Median age was 75 (66–80) years, 50% were women. The median improvement in TCT within 9 days was 25.5 points (=25.5%) (interquartile range [IQR] 12.5–42.5) in the Vojta group and 0 (IQR 0–13) in the control group (*p* = 0.001). Patients treated with Vojta therapy achieved a greater improvement in the MESUPES than patients in the control group (20% vs 10%, *p* = 0.006).

**Conclusion:**

This first randomised controlled trial of Vojta therapy in acute stroke patients demonstrates improvement of postural control through Vojta therapy compared to standard physiotherapy. Although this trial has some methodical weaknesses, Vojta therapy might be a promising approach in early stroke rehabilitation and should be studied in larger trials.

**Trial registration:**

ClinicalTrials.gov; Unique identifier: NCT03035968. Registered 30 January 2017 – Retrospectively registered; http://www.clinicaltrials.gov.

## Introduction

Unilateral motor weakness is one of the most common deficits resulting from stroke and one of the main causes of disability [1, 2]. Scientific evidence demonstrating the values of specific interventions for recovery after stroke is limited and comparisons between different methods in current use have so far failed to show that any particular physiotherapy or stroke rehabilitation strategy is superior to another [3]. Only a few studies address the question of the optimal physiotherapy in stroke rehabilitation. Furthermore, stroke recovery is heterogeneous in terms of outcome [4].

Vojta therapy, also referred to as the Vojta method or reflex-locomotion according to Vojta, is a specialised type of physiotherapy that was discovered and developed by the Czech neurologist and child neurologist Václav Vojta. In the 1960s Vojta observed that maintained peripheral pressure stimulation evoked a stereotypic widespread motor response, as a pattern of tonic muscle contractions in both sides of the neck, trunk and limbs as a result of spatial summation that lead to improvement of postural control [5]. Based on the principles of ontogenetic development Vojta defined postural regulation as the control of body posture and uprighting of the body against gravity as elementary components of locomotion, as well as target-oriented movements of the limbs. To accomplish postural control the individual requires plans and programs (“innate patterns”) that assemble task-related automatic adjustment of movements and posture [6]. The basic principle of Vojta therapy is the regulation of posture [7], which is attained within the innate movement sequences of reflex-locomotion, is retrievable at all times and can be found in all forms of human locomotion representing the basis for human movement [5–7]. To activate these innate patterns of movement, the therapist applies pressure to defined zones. There are ten zones distributed over the trunk, the arms and legs (e.g. aponeurosis of the musculus gluteus medius, epicondylus medialis femoris, spina iliaca anterior superior, margo medialis scapulae, chest zone between the 7th and 8th rib) [5, 8, 9]. Vojta therapy is a standardised therapy with defined starting positions (prone, supine or side lying position) and specific initial angular positioning of the trunk and the extremities.

The mechanisms or neurobiological basis underlying the observed effects of Vojta therapy is still not fully understood. Vojta supposed that there might be a phylogenetic old „locomotion-centre “coordinating single responses situated below the upper brainstem. Supported by randomised controlled trials using functional magnetic resonance imaging (MRI), the pontomedullary reticular formation is speculated to play a key role in Vojta therapy and is suggested to participate in locomotor control as well, as it is implicated in anticipatory postural control before gait initiation, by integrating descending cortical influences and ascending spinoreticular inputs [10–12]. Vojta therapy has been reported to activate the trunk musculature, thereby enhancing postural control ability [7, 13, 14]. Originally, Vojta therapy was applied mostly to infants with movement disturbances or known motor disorders. Nowadays, Vojta therapy is used less frequently for adults, therefore clinical evidence is scarce, and it has never been investigated in stroke patients. There are parallels between motor recovery after stroke (relearning) and the acquisition of skilled movement patterns in human infants (innate/learning) [15–17], so that cortical reorganisation after brain injury due to stroke can be compared to those occurring during ontogenetic development [18]. Postural disorders are among the most prevalent consequences after stroke [19]. Until now Vojta therapy is a mostly unknown therapy to stroke physicians. In our hospital we introduced Vojta therapy in 2013 and observed impressive effects in individual acute stroke patients (please refer to videos examples, Additional files 1, 2 and 3). Therefore, we decided to explore the method more deeply by performing a randomised controlled proof-of-concept study (for more details concerning rationale please refer to Additional file 3).

The aim of the trial was to investigate Vojta therapy in acute stroke patients with severe hemiparesis within 72 h after onset, in order to study the potential benefits and possible negative effects of Vojta therapy as a new approach in stroke recovery. We hypothesise, that Vojta therapy compared to standard physiotherapy improves postural control and motor function in early rehabilitation of patients with acute stroke.

## Methods

### Study design

We designed an investigator-initiated, prospective, single-centre, randomised controlled pilot trial conducted in a tertiary academic centre in Frankfurt, Germany. The trial was performed according to good clinical practice and was consistent with the principles stated in the Declaration of Helsinki in 1964 and subsequent revisions. The trial protocol was approved on October 14, 2015 by the ethics committee of the Hessian state chamber of physicians, Germany (reference number FF88/2015). No changes to the protocol were made since the start of the trial.

Written informed consent was obtained from all patients before trial participation. The study protocol is available (Additional file 4). The trial was registered at ClinicalTrials.gov (NCT 03035968).

### Participants

Inclusion criteria were age ≥ 18 years, a confirmed (cranial computed tomography or MRI proven) acute ischaemic or haemorrhagic stroke, randomisation within 72 h after onset of symptoms or after a patient was last seen normal, presence of a severe hemiparesis (medical research council scale for muscle strength of the arm [MRCS] ≤2) [20], a pre-morbid modified Rankin Scale (mRS) [21] with a score ≤ 3, a maximum National Institute of Health Stroke Scale (NIHSS) [22] score of 25, and written informed consent by the patient. Treatment with recombinant tissue plasminogen activator (rtPA) and thrombectomy before randomisation was allowed.

Exclusion criteria were severe cognitive impairment or limitation in communication skills due to aphasia, deteriorated consciousness or dementia (prohibiting comprehension and implementation of the physiotherapy), participation in another clinical trial, and pregnancy.

### Randomisation and masking

Patients were randomly assigned (1:1) with sealed, opaque and sequentially numbered envelopes to receive usual stroke unit care with Vojta therapy (intervention group) or standard physiotherapy (control group). Investigators were instructed to assign the patient according to the envelope with the lowest randomisation number. Randomisation was done by the investigator, who enrolled the subject. Blinding of the primary outcome - improvement of postural control at day 9 - was not feasible in the current setting of this pilot trial. Secondary clinical outcomes – severity and quality of disability after 3 months – were done by a physician who was blinded to the treatment.

### Procedures

The control group received conventional physiotherapy which consisted of repetitive sensoriomotor exercises using existing function of the affected extremity in task-oriented training and movements used during daily activity, passive movements of the limbs, trunk strengthening exercises, goal directed movements and mobilisation including gait training.

The intervention group received Vojta therapy (Video example: Additional file 5). Vojta therapy was implemented by stimulating the chest zone, which is located between the 7th and 8th rib, use of additional zones was allowed to support the activation. The starting positions for Vojta therapy were supine or side-lysing position with the head turned 30°toward the side being stimulated or side-lying. Patients were treated for 30 min with Vojta therapy and afterwards were mobilised with gait training, if feasible.

Both groups received one treatment sessions daily consisting of 40 min. Seven sessions in total were given to all patients in both groups before the primary outcome assessment, so that both groups had the same amount of therapy. All physiotherapists treating the intervention group had received special training and certification from the International Vojta Society (please refer to Additional file 3). No Vojta-therapist treated patients in the control group. All staff enrolled in this study were trained for the assessments by the main investigator and the principal physical therapist.

### Outcome assessments

An overview of the trial schedule (interventions and visits) is provided in Table 1. The *Trunk Control Test (TCT)* [23–27] *–* the measurement of the primary endpoint - was assessed at day 2 after admission to the hospital before the first treatment (baseline), at day 5 and day 9 after treatment. The TCT is a validated test to assess motor impairment and postural control after stroke. A range of 0 (patient is not able to roll at all from a supine position) to 100 (patient is able to sit for 30 s independently on the edge of the bed) points can be achieved (Additional files 3 and 6) Secondary clinical outcomes were measured with the following scales: The *NIHSS* [22, 28] was used as a quantitative measure of neurological deficit in stroke patients. The *Catherine Bergego Scale (CBS)* [29, 30] is a standardised checklist to assess the presence and extent of neglect in patients with stroke and hemispatial neglect. The *Motor Evaluation Scale for Upper Extremity in Stroke Patients (MESUPES)* [31, 32] is a clinical and research tool to qualitatively evaluate arm and hand function during recovery after stroke. The *mRS* [21, 33] is an ordinal scale for functional independence with reference to pre-stroke activities. The *Barthel Index (BI)* [34, 35] is a validated measure of disability.

**Table 1:** Trial schedule on interventions and outcome visits

	Visit 1 (day^a^ 2 [+/− 1]) (before therapy)	Visit 2 (day^a^ 5 [+/− 1])	Visit 3 (day^a^ 9 [+/− 1]) (after therapy)	Visit 4 (day^b^ 90[+/− 5]) (phone contact)
**TCT**^**d**^	x	x^**e**^	x	
**NIHSS**	x		x	
**CBS**	x^c^	x^c^	x^c^	
**MESUPES**	x^c^	x^c^	x^c^	
**mRS**	x		x	x
**Barthel Index**	x		x	x

*TCT* = Trunk Control Test, *NIHSS*=National Institutes of Health Stroke Scale, *CBS*=Catherine Bergego Scale, *MESUPES* = Motor Evaluation Scale for Upper Extremity in Stroke Patients, *mRS* = modified Rankin Scale

^a^ day after admission to hospital

^b^ day after stroke onset

^c^ Assessment before and after therapy

^d^ primary outcome measure

^e^ Assessment only after therapy


**Additional file 6: Video 4**. Video example of the Trunk Control Test (TCT). The video demonstrates the different tasks of the Trunc control test. (WMV 11231 kb)


Assessments were performed by two physiotherapists (one from the Vojta- and one from the control group) following the four-eyes principle. In case of disagreement in scoring, a third physiotherapist conducted an additional assessment. Full details of the outcome assessments TCT, NIHSS, CBS, MESUPES, mRS, BI and the MRCS (used for screening only) are given in Additional file 3.

### Outcome measures

The primary outcome was defined as improvement of postural control measured by the difference of scores in the TCT at baseline and day 9. We chose the primary outcome at 9 days, firstly, because this is the mean of the longest duration of stay of these patients at our hospital and secondly, because Vojta therapy is not available in most rehabilitation clinics.

Secondary outcomes included an improvement in degree of neglect (measured with the CBS), the arm motor function (measured with MESUPES), and clinical improvement of stroke severity (measured with NIHSS) and disability (measured with the BI) on day 9 after admission compared to baseline, as well as an improvement of the mRS and the BI on day 90 after stroke onset compared to baseline. The 90 day mRS and BI were assessed via telephone interview by a blinded assessor.

Adverse events (AE) and serious adverse events (SAE) during the hospital stay and all deaths and SAE until day 90 were recorded and assessed by the investigators according to standard definitions. All AE and SAE were evaluated and forwarded to a medical expert for assessment of relatedness to the study treatment.

### Sample size calculation and statistical analysis

A sample size calculation for this pilot trial was not feasible, because desired parameters, such as the estimated effect size or the standard deviation due to missing prior information or data in the literature could not be estimated. For feasibility reasons we chose a sample size of 40 subjects (20 per group) in order to complete recruitment in this monocentric trial within 1,0 to 1,5 years.

Analyses were based on intention to treat (ITT). Normal distribution of the variables was tested by Shapiro-Wilk-test. Tested variables predominantly did not show a normal distribution (*p* < 0,05). Therefore, for comparison of the samples we used non-parametric tests for non-normal distributed samples. Variables with non-Gaussian distribution are expressed as median (interquartile range [IQR]) and qualitative variables as number (%). Categorised data were analysed with the Fisher’s exact test. Changes from baseline to day 9 in TCT score (primary outcome) between the two groups were compared by Mann–Whitney-U-test. Similar analyses were made for the CBS, MESUPES, NIHSS, mRS and BI parameters between day 90 and baseline.

We also performed an analysis of variance (ANOVA) and an analysis of covariance (ANCOVA) (in case of continuous factors such as age and NIHSS) to test the influence of potentially confounding factors (age, sex, NIHSS at baseline, stroke side, performance of thrombectomy or thrombolysis) on outcome and interaction with treatment. All tests used SPSS for Windows (version 24.0 SPSS Inc., USA) and R for Windows (version 3.5.0). All tests were two-sided and *p* values < 0.05 were considered statistically significant.

## Results

**Fig. 1:**
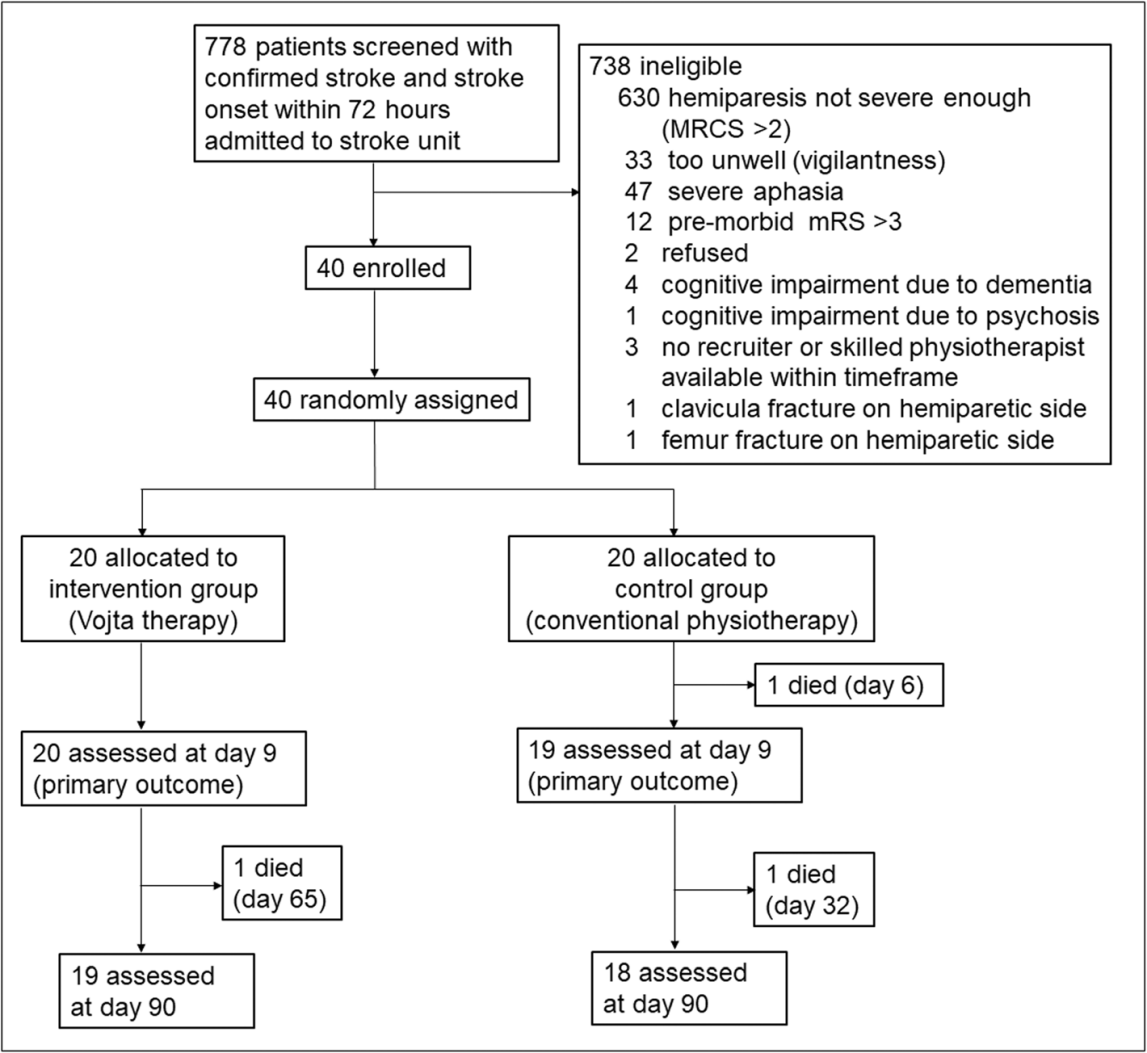
Consort diagram on trial flow. MRCS = medical research council scale for muscle strength; mRS = modified Rankin Scale

We screened 778 patients and randomly assigned 40 patients to receive either Vojta therapy (*n* = 20) or standard physiotherapy (*n* = 20) between December 02, 2015 and July 05, 2017. Figure 1 shows the trial profile. One patient in the control group died at day 6, before reaching the primary outcome due to a malignant middle cerebral artery (MCA) infarction. Thirty-nine patients (97.5%) were included in the ITT analysis. Two patients (one in each group) died after discharge from hospital before day 90, one due to pneumonia in the rehabilitation centre on day 65 (Vojta group) and one due to endocarditis on day 32 (control group). Thirty-seven patients (92.5%) were included in the three-months follow-up assessment.

The median age of patients was 75 (66–80) years, and 20 (50%) were women. No patient was lost to follow-up until study end. Baseline characteristics did not differ statistically between the two groups, with the exception of the side of lesion, prior known orthopaedic conditions, and scores in TCT, CBS and NIHSS at baseline (Table 2), so that patients randomised to Vojta group were slightly more affected by the stroke. The TCT baseline scores differed noticeably between both groups (Vojta group 25 [0–43] vs control group 56 [42.5–87]).

**Table 2:** Demographics and baseline clinical characteristics

	Vojta group (*n* = 20)	Control group (*n* = 20)
Sex		
Female	11 (55%)	9 (45%)
Male	9 (45%)	11 (55%)
Age, median (IQR)	77 (72.5–85)	72.5 (64–78)
Risk factors		
Hypertension	15 (75%)	16 (80%)
Diabetes mellitus	5 (25%)	3 (15%)
Smoker^a^	3 (15%)	4 (20%)
Artrial fibrillation	4 (20%)	3 (15%)
Orthopaedic disease^b^	9 (45%)	1 (5%)
Right handedness	20 (100%)	19 (95%)
Prior stroke	5 (25%)	7 (35%)
Premorbid history		
Premorbid mRS, median (IQR)	0 (0–2)	0·(0–1.5)
Premorbid mRS, mean (SD)	0.9 (1.25)	0.65 (1.09)
Walking without aid	14 (70%)	16 (80%)
Stroke history		
Ischaemic stroke	20 (100%)	19 (95%)
Haemorrhagic stroke	0 (0%)	1 (5%)
Left MCA infarct	2 (10%)	8 (42%)
Right MCA infarct	17 (85%)	10 (53%)
Brainstem infarct	1 (5%)	0 (0%)
Other or multiple locations^c^	0 (0%)	1 (5%)
NIHSS at admission	14.5 (12–16)	12 (8.5–15)
rtPA treatment administered	8 (40%)	5 (26%)
Thrombectomy performed	7 (35%)	5 (26%)
Patients with neglect	16 (80%)	11 (55%)
Scores at baseline, median (IQR)		
Trunk control test	25 (0–43)	56 (42.5–87)
MESUPES	2 (0–5.0)	3 (1–10.5)
CBS	4 (3–5.5)	3 (2–4)
modified Rankin Scale	5 (4–5)	4 (4–5)
Barthel Index	3 (0–3.5)	5 (1–9)
NIHSS after randomisation	15 (12.5–16.5)	11.5 (10–14.9)

Data are n (%) or median (interquartile range, IQR)

*mRS* modified Rankin Scale, *MCA* middle cerebral artery, *rtPA* recombinant tissue plasminogen activator, *MESUPES* Motor Evaluation Scale for Upper Extremity in Stroke Patients (part 1 to 4 = MESUPES-arm), *CBS* Catherine Bergego Scale (part 5 and 6), *NIHSS* National Institutes of Health Stroke Scale, *SD* standard deviation

^a^smoker defined as current smoker or quit smoking in the last past 2 years. ^b^Prior known orthopaedic diseases: hip or knee arthroplasty, coxalgia, gonarthrosis, surgery after lumbar disc hernation, sciatic pain syndrome and stenosis of the spinal canal. ^c^both MCA territories, right posterior territory and left cerebellum

**Fig. 2:**
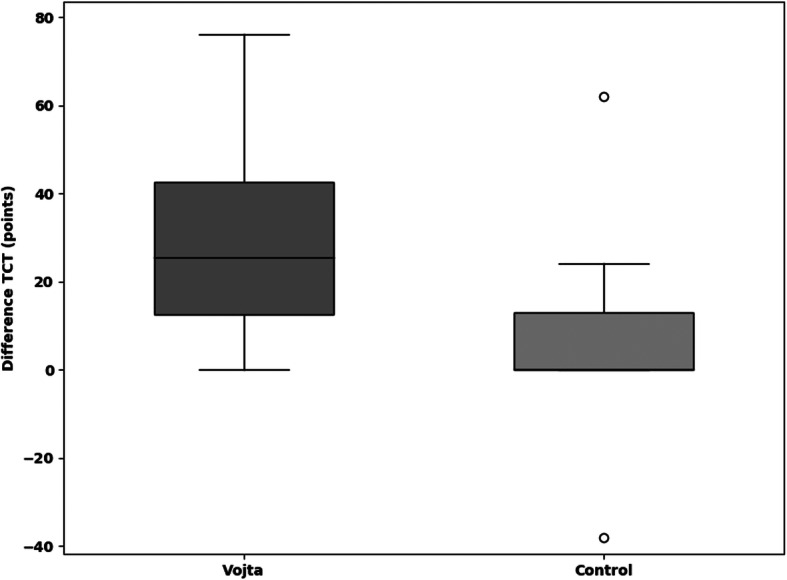
Difference in Trunc control test (TCT) scores between baseline and day 9. The median improvement in the TCT within 9 days (primary outcome) was 25.5 points (=25.5%) (IQR 12.5–42.5) in the Vojta group and 0 (IQR 0–13) in the control group (*p* = 0.001). Data are presented as box-and-whisker plots, in which the top and bottom of the rectangles indicate the 75th and 25th percentiles, respectively; the horizontal lines within the rectangles indicate the 50th percentile (median); the lines above and below the rectangles indicate the minimum and maximum of all of the data, so far as this are no outlier. Outlier are data lying outside the box > 1,5 lenghts of the box (IQR) and are presented as a small circle

**Fig. 3:**
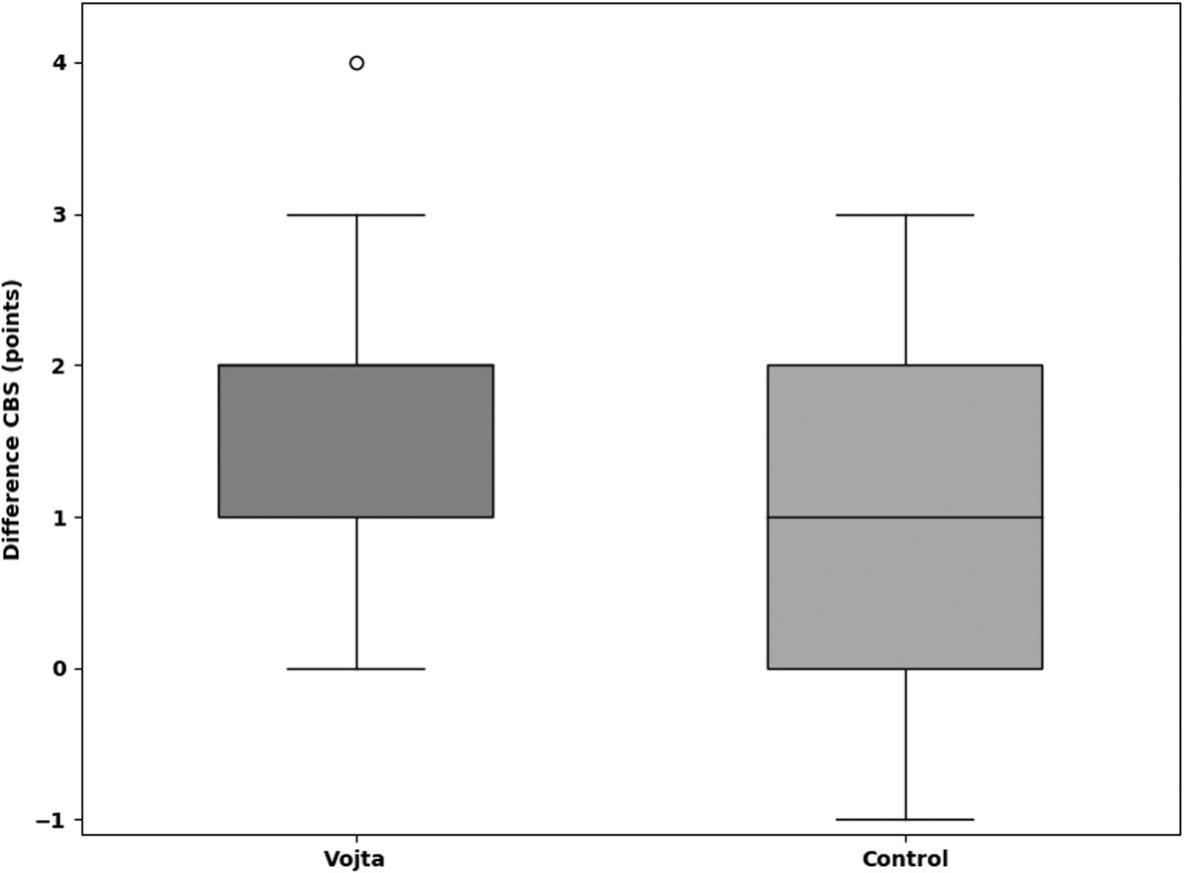
Difference in Catherine Bergego Scale (CBS) scores between baseline and day 9. The median improvement in the CBS (Neglect test) within 9 days was 2 points (=33.3%) (IQR 1–2) in the Vojta group and 1 point (=16.6%) (IQR 0–2) in the control group (*p* = 0.054). Data are presented as box-and-whisker plots

**Fig. 4:**
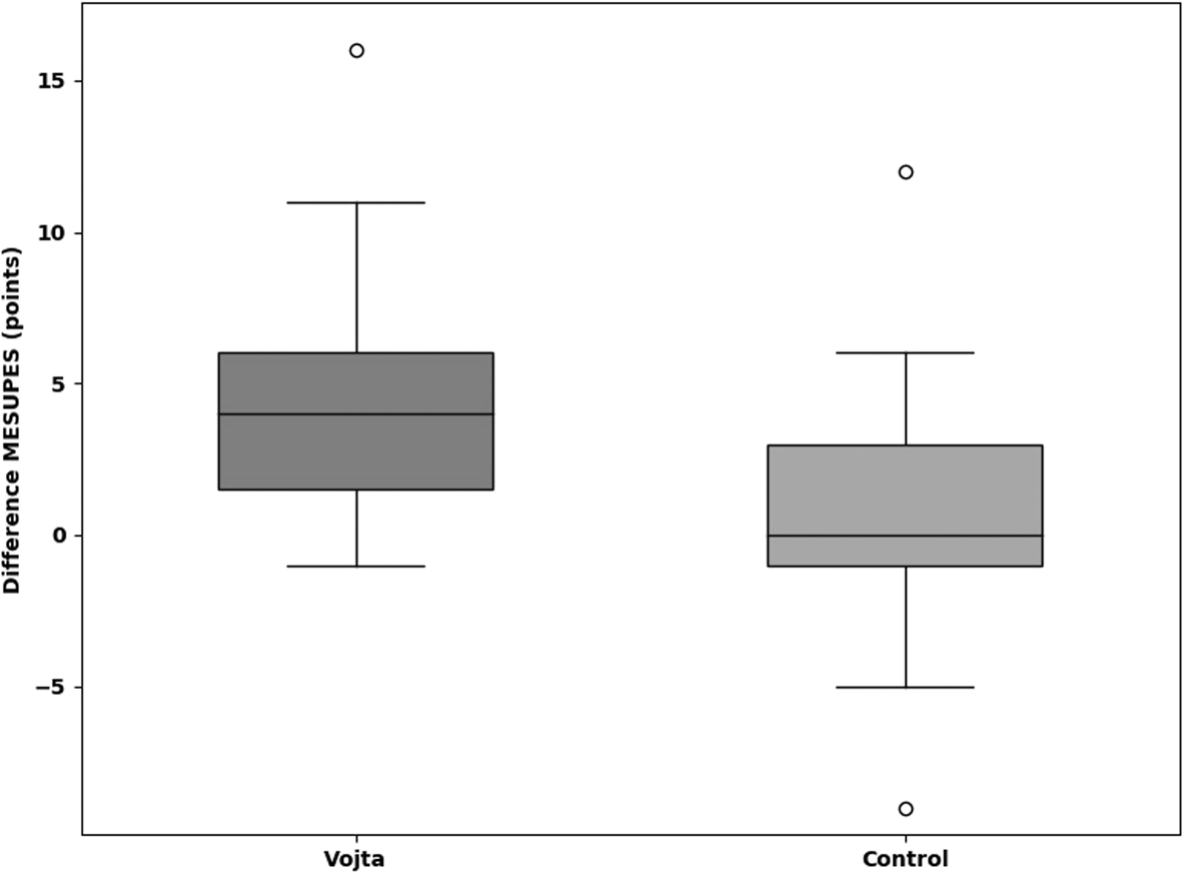
Difference in motor evaluation scale for upper extremity in stroke patients (MESUPES) scale scores between baseline and day 9. The median improvement in the MESUPES within 9 days was 4 points (=20%) (IQR 1.5–6) in the Vojta group and 2 points (=10%) (IQR 0–5) in the control group (*p* = 0.006). Data are presented as box-and-whisker plots

**Fig. 5:**
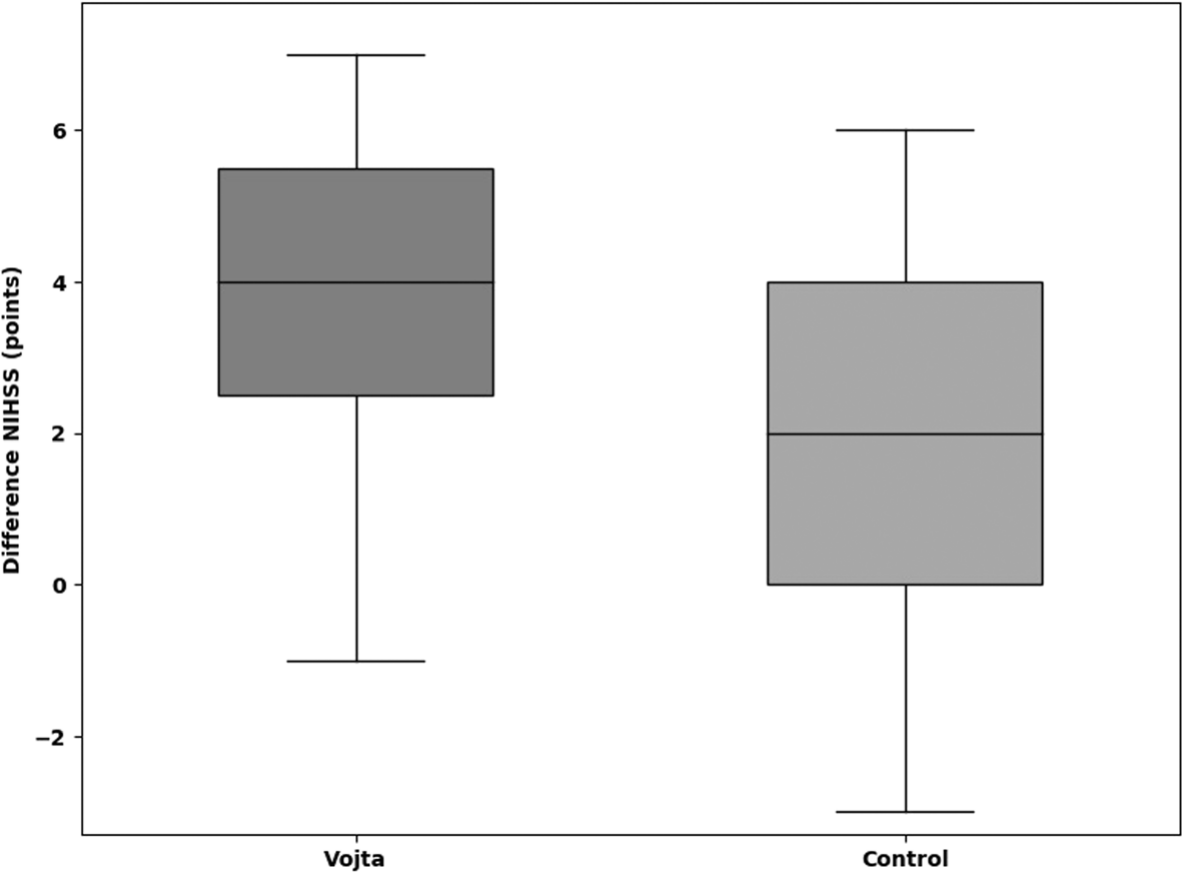
Difference in National Institutes of Health Stroke Scale (NIHSS) scores between baseline and day 9. The median improvement in the NIHSS within 9 days was 4 points (=9.5%) (IQR2.5–5.5) in the Vojta group and 2 points (= 4.8%) (IQR 0–4) in the control group (*p* = 0.022). Data are presented as box-and-whisker plots

The median improvement in the TCT within 9 days (primary outcome) was 25.5 points (=25.5%) (IQR 12.5–42.5) in the Vojta group and 0 (IQR 0–13) in the control group (*p* = 0.001) (Fig. 2**,** Additional file 7). In the CBS (neglect test) the Vojta group showed a median improvement of 33.3, and 16.6% in the control group (*p* = 0.054) (Fig. 3**,** Additional file 7). Vojta patients achieved a greater improvement in the MESUPES (*p* = 0.006) and the NIHSS (*p* = 0.022) than patients in the control group (20% vs 10%, and 9·5% vs. 4·8%, respectively) (Figs. 4 and 5 and Additional file 7). There was a trend showing greater improvement in the BI from baseline to day 9 in the Vojta group (17.5% in Vojta group, 10% in control group; *p* = 0.083) (Additional file 7).

At 90 days the median mRS score was 4 (3–4.5) in the Vojta group and 3.5 (3–4) in the control group. The BI showed a non-significant improvement of 30% in the Vojta group and 22.5% in the control group from baseline to day 90 (Additional file 7).

For the primary outcome we investigated possible influencing factors. Neither the NIHSS at randomisation, age, sex, side of infarction (right or left sided MCA infarction; *n* = 37), nor administration of rtPA or performance of thrombectomy showed a statistically significant influence on TCT results. ANOVA and ANCOVA considerations showed no interactions between these factors and treatment, but revealed the treatment effect of Vojta therapy compared to conventional physiotherapy.

The total number of SAEs until day 9 was 25 (Additional file 8), the number of patients with SAE was similar in each group: 12 in the Vojta group and 13 in the control group. No SAE was considered by the investigators to be related to treatment.

## Discussion

We compared two physiotherapeutic approaches implemented within 72 h after acute stroke and studied their effect on trunk control. Vojta therapy significantly improved postural control and motor function compared to conventional therapy in patients with acute stroke within 9 days of symptom onset. Furthermore, this pilot study demonstrated that Vojta therapy is feasible, and safe in very early stroke rehabilitation. We did not see a significant difference in clinical outcomes at day 90, but the trial was not designed for this outcome.

The selection of a primary outcome parameter is a challenge in every clinical trial, especially in trials on recovery, with many different assessment tools used in RCTs [36]. We chose the TCT as a primary outcome parameter, because it was designed to measure postural control which is an important prerequisite for further mobility. Early control of sitting balance as tested with the TCT and as basic requirement for regaining standing balance and later gait is regarded as an important factor for the final outcome at 6 months [4, 37]. Furthermore, the TCT can be used to test postural control in bedridden stroke patients and has an excellent interrater reliability (*r* = 0.76, *p* < 0.001) [23, 27]. A disadvantage of the TCT is the ceiling effect in patients with a higher grade of independence in activities of daily living, as they can achieve maximum scores in any of the four test items, although they may still have motor deficits. The ceiling effect was proven in a cohort of non-acute and chronic stroke patients [27], but probably this psychometric characteristic of measurement also occurs in acute stroke populations. In our trial the ceiling effect may have affected the control group to a greater extent, because the control group had higher median scores at baseline (intervention 25 [0–43] vs control 56 [42.5–87]). Improvement in the TCT was significantly greater in the Vojta group than the control group, but imbalance at baseline and the ceiling effect have to kept in mind when interpreting these results.

Only a few recovery trials have initiated restorative treatments within 7 days after onset and can therefore be classified as acute [38]. Delays to the initiation of rehabilitation seem to be associated with a poorer outcome and a longer length of stay in hospital for patients [39]. Furthermore, motor training started around 5 days after stroke is more effective than training started at day 14 or day 30 [40]. The fact that there is a limited time window for the greatest motor recovery and increased receptivity to training regimes after injury is congruent with observations in animal models suggesting that there is about a month of heightened plasticity in the brain early after stroke when most recovery from impairment occurs [4, 18]. For this reason, we chose to start treatment within 72 h after stroke onset in our trial.

### Limitations and strengths

This trial is, to our knowledge, the first randomised controlled trial (RCT) of Vojta therapy in acute stroke patients. No patient was lost to follow up. We documented the time of intervention, stroke onset and provide detailed information about the training regimes as requested by the Stroke Recovery and Rehabilitation Roundtable taskforce [38]. Furthermore, we present detailed baseline characteristics, to enhance interpretation of the results [36, 41]. Age and stroke severity are the strongest predictors of outcome after acute stroke [42], and even if the Vojta group was slightly more disadvantaged in regard to both the NIHSS at baseline (15 in Vojta group and 11.5 in control group) and age (77 years vs 72.5), the Vojta group showed a greater clinical improvement. Our trial is one of relatively few trials of recovery within the first week after stroke onset [38, 43].

This trial has several limitations: Firstly, the sample size was small, as it was a single centre pilot trial and was therefore not powered to compare the effect on clinical outcomes at 90 days. Secondly, investigators and therapists were not blinded to the treatment allocation while clinical assessment at day 90 was blinded. The primary and secondary outcome assessments were done by two physiotherapists (one from each treatment group) to ensure some control. All study personnel received special training before study initiation. The TCT is easy and clear to test. We also chose the TCT, because evaluation is explicit: for each item involving axial movements points are only given, if the patient achieves the complete task (i.e. sitting-up from a lying position – yes or no). The tester is not allowed to assist at all, so that subjectivity is reduced to a minimum. Nevertheless, we cannot exclude an observer bias and we have to point out that unblinding on the primary outcome measure is our crucial limiting factor. Thirdly, we noted some imbalances in baseline values, but whereas the differences concerning side of lesion, prior known orthopaedic conditions and age seemed unlikely to be relevant with regards to the primary outcome, the baseline difference in TCT possibly had a relevant impact on the results, especially the primary endpoint. Fourthly, there is a selection bias due to the exclusion of patients with severe aphasia (*n* = 47), who had to be excluded from the study, because of the foreseeable non-compliance. For this reason, we had to exclude more patients with a left hemispheric infarct, which is reflected by the stroke localisation of the study cohort and may therefore not be completely generalisable to a usual stroke unit population. We limited the application of Vojta therapy to the hospital stay, because this physiotherapeutic approach is not common in stroke rehabilitation. In summary, the possible observer bias due to unblinded primary outcome measure, the psychometric problems of the outcome measure (TCT ceiling effect) and the difference in baseline scores of the TCT are relevant methodical flaws and we can not estimate how much these factors influenced the results, therefore the results have to be evaluated with caution.

## Conclusion

In this first pilot trial, treatment with Vojta therapy was beneficial in the early rehabilitation of acute stroke patients with a severe hemiparesis within 72 h after onset showing improved postural control, degree of neglect and motor function compared to standard physiotherapy, keeping the methodical weakness of the trial in mind. Due to cost savings in many countries we should also take into consideration, that only some patients go on to receive an inpatient rehabilitation (if existent at all) [38]. For many patients access to a multidisciplinary rehabilitation team is only possible in the stroke unit and therefore the focus on acute stroke recovery seems to be even more important. Vojta therapy is an economical approach and has the advantage, that relatives can be trained in the basic treatment techniques to continue Vojta therapy after discharge. The results of this trial have to be verified by a multicentric RCT with a larger sample size and a blinded primary outcome assessment.

## Supplementary information

**Additional file 1: Video 1.**. Video example of motor function before Vojta therapy. Female patient with left middle cerebral artery infarction 6 months ago with a right-sided hemiparesis, who came to our physiotherapy centre. After the stroke (in the sub-acute stage) she underwent an in-patient and thereafter out-patient treatment in a neurological rehabilitation centre for 6 months. Video 1 demonstrates her arm mobility before the first Vojta treatment (but 6 months after stroke and after other treatment approaches). The patient was asked to touch her mouth with her fingers but is not able to perform this task. She then received Vojta therapy for 30 min.

**Additional file 2: Video 2.**. Video example of motor function after Vojta therapy. In video 2 the patient performed the same task (touch the mouth) after 30 min of Vojta therapy. She performs much better, due to improved postural control. Both videos (1 and 2) were filmed the same day, before and after the first Vojta treatment.

**Additional file 3.** Supplementary Material. Supplementary background information concerning Vojta therapy, rationale for the trial and supplementary information on methodology (details concerning TCT, therapeutic intervention and staff qualification or outcome assessments).

**Additional file 4.** Study Protocol Vojta Trial (2015). Original study protocol as published in Clinical Trials.gov and institutional homepage.

**Additional file 5: Video 3.**. Video example of Vojta therapy. Video demonstration of Vojta therapy. The patient is in a side lying position. The therapist activates the “chest zone” by applying finely graded pressure to this zone and gives a guiding resistance to the head to support the activation. These stimuli lead to a reflexive movement of the limb in this adult patient as is seen in newborns/babies where Vojta therapy is also implemented. In other words, the stimulus reproducibly provokes limb movements and does not require active willed cooperation. The patient’s right arm is activated and later also the leg.

**Additional file 7.** Table of test results. Results of all tests for primary and secondary outcomes are provided in this table.

**Additional file 8.** Adverse events during hospital stay. List of all adverse events during hospital stay (until discharge).

## Data Availability

The datasets used and/or analysed during the current study are available from the corresponding author on reasonable request.

## Abbreviations

AE: Adverse events
ANOVA/ANCOVA: Analysis of (co)variance
BI: Barthel Index
CBS: Catherine Bergego Scale
CCT: Cranial computed tomography
fMRI: Functional magnetic resonance imaging
ITT: Intention to treat
IQR: Interquartile range
MESUPES: Motor Evaluation Scale for Upper Extremity in Stroke Patients
MRCS: Medical research council scale for muscle strength
mRS: Modified Rankin Scale
NIHSS: National Institute of Health Stroke Scale
RCT: Randomised controlled trial
rtPA: Recombinant tissue plasminogen activator
SAE: Serious adverse events
SD: Standard deviation
TCT: Trunk Control Test
