# Supplementary material for: Vojta therapy improves postural control in very early stroke rehabilitation: a randomised controlled pilot trial
Source: Neurol Res Pract. 2020 Aug 20;2:23. doi: 10.1186/s42466-020-00070-4 (PMC7650119; doi:10.1186/s42466-020-00070-4)
Supplement: Supplementary file 4 — Additional file 3. Supplementary Material. Supplementary background information concerning Vojta therapy, rationale for the trial and supplementary information on methodology (details concerning TCT, therapeutic intervention and staff qualification or outcome assessments). [file 42466_2020_70_MOESM3_ESM.pdf]

SUPPLEMENTARY MATERIAL

NEUROLOGICAL RESEARCH AND PRACTICE

Vojta therapy improves postural control in very early stroke rehabilitation: A randomised controlled pilot trial

Content:

1. Video examples
    1.1 Videos 1 and 2: Video example of motor function before and after Vojta therapy
    1.2 Video 3: Video example of Vojta therapy
    1.3 Video 4: Video example of the Trunk Control Test
2. Supplementary background material
    2.1 Vojta therapy and Vojta training
        2.1.1 Vojta therapy
        2.1.2 Vojta training and certification
    2.2 Rationale for the study
        2.2.1 Why Vojta therapy in stroke recovery?
        2.2.2 Clinical observations
3. Supplementary material on methodology
    3.1 Therapeutic intervention and staff qualification
        3.1.1 Therapy for the intervention group
        3.1.2 Therapy for the control group
    3.2 Outcome assessments
4. Supplementary references

1. Video examples

1.1 Video example of motor function before and after Vojta therapy

Female patient with left middle cerebral artery infarction 6 months ago with a right-sided hemiparesis, who came to our physiotherapy centre. After the stroke (in the sub-acute stage) she underwent an in-patient and thereafter an out-patient treatment in a neurological rehabilitation centre for 6 months.

Video 1 (**Additional file 1**): The first video demonstrates arm mobility before the first Vojta treatment (6 months after stroke and after other treatment approaches). The patient was asked to touch her mouth with her fingers but is not able to perform this task. She then received Vojta therapy for 30 minutes.

Video 2 (**Additional file 2**): In the second video the patient performed the same task (touch the mouth) after 30 minutes of Vojta therapy. She performs much better, due to improved postural control.

Both videos were filmed on the same day before and after the first Vojta treatment.

1.2 Video example of Vojta therapy

Video 3 (**Additional file 5**): Demonstration of Vojta therapy. The patient is in the side lying position. The therapist activates the "chest zone" by applying a finely graded pressure to this zone and gives a guiding resistance to the head to intensify the activation. These stimuli lead to an involuntary active (reflexogenic) movement of the limb in this adult patient as seen in newborns/babies where Vojta therapy is also implemented. In other words, the stimulus reproducibly provokes limb movements and does not require active willed cooperation of the patient. The patient's right arm is activated and later also the leg.

1.3 Video 4: Video example of the Trunk Control Test (TCT) (primary endpoint)

Video 4 (**Additional file 6**): The video demonstrates the different tasks of the Trunk Control Test. The TCT examines 4 items of axial movements:

1) rolling from a supine position to the affected side;
2) rolling from a supine position to the non-affected side;
3) sitting up from a lying-down position; and
4) sitting in a balanced position on the edge of the bed with feet off the ground for 30 seconds.

Each item can be scored 0, 12 or 25 points:

- 0 unable to perform movement without assistance;
- 12 able to perform movement, but in an abnormal style (Examples: pulls on bed clothes or monkey pole, or uses arms to steady self when sitting);
- 25 able to complete movement normally.

For the sitting balance item, a patient scores 12 if he needs to touch anything with his hands to stay upright, and 0 if he is unable to stay up (by any means) for 30 seconds. The TCT score is the sum of the scores obtained on the four tests and ranges from 0 (minimum) to 100 (maximum and indicating better performance). The examiner's score must relate solely to the performance during the test.

2. Supplementary background material

2.1 Vojta therapy and Vojta training

2.1.1 Vojta therapy

Vojta therapy is a type of physiotherapy, also called the Vojta method. Václav Vojta was a Czech neurologist and child neurologist[1]. In children with cerebral palsy Vojta observed recurring motor reactions in the trunk and the extremities in response to certain stimuli (finely graded pressure applied to specific zones) in combination with specific positioning of the body. He could provoke coordinated muscle activity and patterns of movements that were normally not available to the patient and discovered, that children with cerebral palsy treated with this method exhibited better gait, better posture and better speech after therapy [1, 2].

---

[1] He was born on the 12th July 1917 in Mokrosuky, Bohemia, the Czech Republic. He died on the 12th September 2000 in Munich. Vojta developed the bases of his diagnostics and therapy, between 1950 and 1970, while searching for treatment possibilities for children and adolescents with cerebral palsy.

Vojta described the innate movement sequences of reflex locomotion that are retrievable at all times and can be found in all forms of human locomotion representing the basis for human movement [1]. He observed that reflex locomotion enables elementary patterns of movement to be restored once more i.e. they become accessible once more - at least partially - in patients with impairment of the central nervous system and locomotor system, assuming that repeated stimulation of these "reflex-provoked" movements can lead to something like "new networking" within functionally blocked neuronal networks.[1] "Reflex", in terms of reflex locomotion, does not refer to the kind of neuronal regulation, but to the provoked predetermined, "automatically" available movement responses as a result of the therapeutically applied external stimuli and specific positioning of the body.

Vojta therapy has been reported to activate the trunk musculature, in particular the abdominal muscles and the intrinsic deep muscles of the spine to regulate trunk stability and increase the potential intersegmental rotation of the spine, thereby enhancing postural control ability [3-5]. More attention has been given recently to the importance of the coordinated activity of the trunk in motor skills in particular gait and functional trunk stability [3, 6-9].These studies support indirectly Prof. Vojta's empirical findings in relation to the finely coordinated muscle interplay and differentiated muscle function seen in reflex locomotion. Vojta therapy has a targeted effect on the regulation of body posture which requires in particular the highly coordinated activity of the ventral lying trunk musculature which demonstrate in part both postural and respiratory functions [2]. This finding was well documented by Prof. Vojta in children with cerebral palsy (CP - athetosis, spasticity). The above-mentioned highly coordinated muscle interplay is disturbed to a greater or lesser extent, in the habituated pathological postural patterns of cerebral palsy or milder postural dysfunction. It is always seen in context to the coordinated activity of the external rotators of both the shoulder and hip joints and in context to the muscle coordination influencing axial extension and intersegmental rotation of the spine. The functional consequence is the ability to control the isolated movements of the pelvis and the head in the erect posture. Vojta therapy has the ability to activate this entire muscle interplay at least segmentally, depending on the severity of the condition.

To activate these innate patterns of movement, the therapist applies targeted pressure to defined zones. There are ten zones distributed over the trunk, the arms and legs (for example aponeurosis of the musculus gluteus medius, epicondylus medialis femoris, spina iliaca anterior superior, margo medialis scapulae, chest zone in the intercostal space between the 7th and 8th ribs) [10-12]. Vojta therapy is a standardised therapy with defined starting positions (prone, supine or side lying position) and specific positioning of the head, trunk and the extremities.

In everyone—regardless of age—such stimuli lead automatically and involuntarily, i.e. without active willed cooperation of the patient, to two movement complexes: *Reflex creeping* in a prone position and *reflex rolling* from a supine and side lying position. Reflex creeping leads to a type of creeping movement, while reflex rolling begins from a supine position, and progresses, via side lying, into crawling.

2.1.2 Vojta training and certification

Certification in Vojta therapy for physiotherapists is possible for two different target groups of patients: Course on Applied developmental kinesiology for babies, children and adolescents with movement disorders according to Vojta ("*Vojta-children's course*") or course on applied developmental kinesiology for adolescents and adults with movement disorders according to Vojta ("*Vojta-adult's-course*"). Prerequisite for the course is two years clinical practice as a physiotherapist. The children's course is 8 weeks in total, the adult's course is 6 weeks in total. Candidates have to deliver a case study to receive the final certificate from the International Vojta Society.

2.2 Rationale for the study

2.2.1 Why Vojta therapy in stroke recovery?

During development the brain shows high plasticity as new connections are formed redundantly and removed through use-dependent processes [13]. There are parallels between motor recovery after stroke (relearning) and the acquisition of skilled movement patterns in human infants (innate/learning) [14], so that cortical reorganization after brain injury due to stroke can be compared to those occurring during physiological development[13]. It is known that the cerebral cortex can reorganize its neural networks[15], but it is unclear how the remapping of lost function is initiated. When the normal input to a particular area of the primary somatosensory cortex is lost because of injury, rapid structural and functional reorganization results in this area being activated by sensory stimulation of the surrounding intact body regions [15]. We propose that Vojta therapy may be such an activating stimulus.

Observations in animal models suggest that there is about a month of heightened plasticity in the brain early after stroke, when most recovery from the impairment occurs [13, 16]. Therefore, there seems to be a limited time window for the greatest motor recovery and increased receptivity to training regimes in stroke rehabilitation. In newborns with motor development disturbances early initiation of a suitable therapy is highly recommended to prevent the appearance of incorrect movement patterns. For the same reason we think it is beneficial to start Vojta treatment as early as possible after a stroke.

2.2.2 Clinical observations

In 2013 we integrated Vojta therapy into the established treatment concept (predominantly Bobath concept) on our stroke unit. We observed that Vojta therapy improved the efficacy of other approaches, working with repetitive exercises that activated automatic movement patterns which led to an improved postural control, uprighting against gravity and target-oriented movements. Directly after Vojta therapy stroke patients showed an improvement in their posture and movement patterns that tended to consolidate after a few days on the stroke unit. As an early effect we observed muscle fasciculations in the motor target area - even in the plegic limbs, appearance of movement sensation in neglect regions, as well as vegetative (pilomotor, vasomotor, sudomotor) reactions in the therapeutic area. Patients reported sensory effects as a heat sensation projected to the periphery, suggesting a systemic effect of Vojta therapy. These clinical observations motivated us to further investigate Vojta therapy in stroke recovery.

We designed a randomised clinical trial (RCT) to compare Vojta therapy and conventional physiotherapy in patients with acute ischemic (AIS) or haemorrhagic stroke (ICH). This RCT is the first trial to investigate improvement of postural control using Vojta therapy in early rehabilitation of stroke patients, which is a very new approach in stroke-rehabilitation. The aim of the trial was to investigate Vojta therapy in acute stroke patients with severe hemiparesis within 72 hours after onset.

3. Supplementary material on methodology

3.1. Therapeutic intervention and staff qualification

3.1.1 Therapy for interventional group

The intervention group received Vojta therapy as physiotherapy. As mentioned above Vojta therapy is defined by starting positions and predefined zones. Primary stimulation was applied to the chest zone, additional use of other zones (see above) in order to support the activation was allowed. The chest zone is located in the intercostal space between the 7th and 8th rib, where the mammillary line crosses the insertion of the diaphragm. The therapist's finger applied a light pressure, progressively oriented in dorsal, medial and cranial directions, toward the opposite scapula. The chest zone was stimulated on the non-paretic side first, and both sides were stimulated at least two times.

The first starting position in our trial was the supine position (**suppl. Fig 1 A**) with the head turned 30° to the side being stimulated and the extremities lying naturally on the bed, then the side lying position (**suppl.Fig 1 B**). The side lying starting position is demonstrated in **Video 3**.

Patients were treated for 30 minutes with Vojta therapy and afterwards were mobilised with gait training, if feasible. Treatment consisted of one daily 40 minutes session with 7 sessions in total for all patients before the primary outcome assessment. All physiotherapists treating the intervention group were certified by the International Vojta Society as advanced training (see http://www.vojta.com). No Vojta-therapist treated patients in the control group.

Supplement Figure 1: Application of Vojta therapy in a patient with right sided hemiparesis:

***A**: Chest zone stimulation in supine position; stimulus given on the non-paretic (left) side*

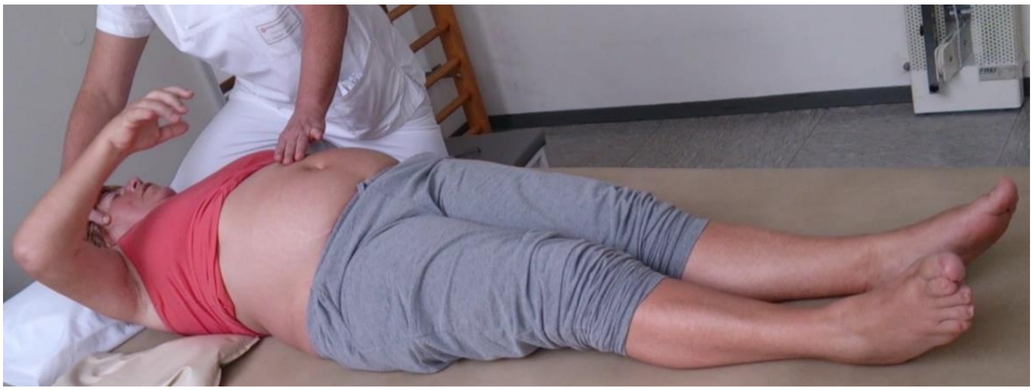

***B**: Chest zone stimulation in side lying position; stimulus given on the non-paretic (left) side*

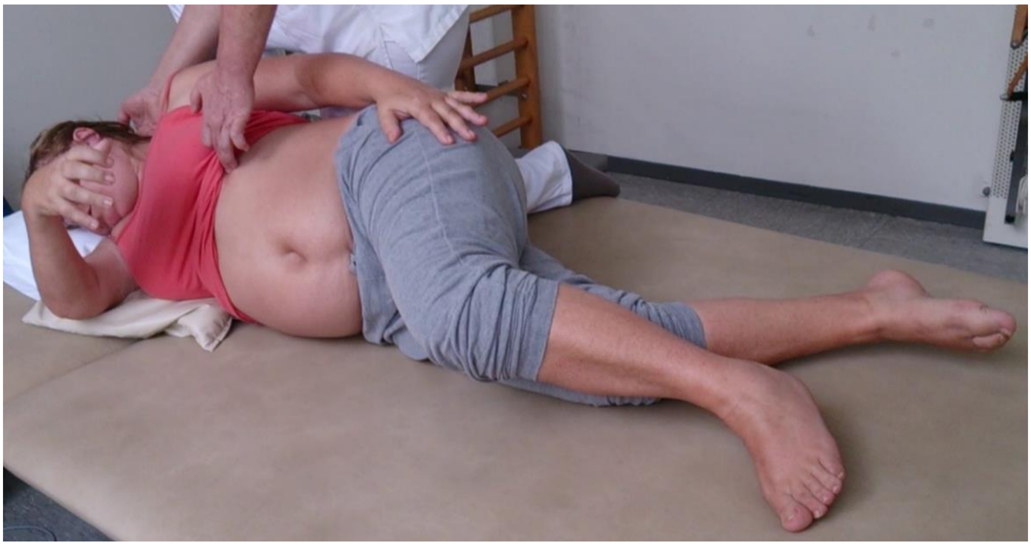

3.1.2 Therapy for the control group

The control group received the standard physiotherapy on our stroke unit ("conventional physiotherapy"). This included repetitive sensorimotor exercises based on task-oriented training with movements used during daily activity, passive movements of the limbs, trunk strengthening exercises, goal directed movements and mobilisation including gait training. Treatment consisted of one daily 40 minutes session with 7 sessions in total for all patients before the primary outcome assessment. Physiotherapists treating the control group were not allowed to be Vojta therapists.

3.2 Outcome Assessments

The *Trunk Control Test (TCT)* [17-21] was assessed at day 2 after admission to the hospital before the first treatment (baseline), at day 5 and day 9 after treatment. The TCT is a validated test to assess motor impairment and postural control after stroke. A range of 0 (patient is not able to roll from a supine position) to 100 (patient is able to sit for 30 seconds independently on the edge of the bed) points can be achieved (**Video 4**).

The *National Institute of Health Stroke Scale (NIHSS)* [22, 23] was assessed at day 2 after admission to the hospital before the first treatment (baseline) and at day 9 after treatment. The NIHSS is used as a quantitative measure of neurological deficit in stroke patients. The 15 items score ranges from 0 to 42, higher scores indicating a more severe neurological deficit and a greater stroke severity.

The *Catherine Bergego Scale (CBS)* [24, 25] and the *Motor Evaluation Scale for Upper Extremity in Stroke Patients (MESUPES)* [26, 27] were assessed at day 2, day 5 and day 9 after admission before and after treatment. The CBS is a standardised checklist consisting of 10 items to assess the presence and extent of neglect in patients with stroke and hemispatial neglect, using a 4-point rating scale for each item (0=no neglect;3=severe neglect). Due to the feasibility of conducting the daily clinical routine and considering the reduced physical resilience of acute stroke patients we shortened the CBS and assessed only item 5 and 6. This resulted in a total score of 6 (0=no neglect, 6=severe neglect). The MESUPES is a clinical and research tool to qualitatively evaluate arm and hand function during recovery after stroke, comprising of 17 items pertaining to arm (8 items) and hand (9 items) performance. The whole test takes up to 30 minutes. In our trial we performed items 1 to 4 of the MESUPES-arm, in order not to overburden the acute stroke patients. Only the first 4 items are performed in a lying position, so that even severely affected patients were able to perform these tasks. The total score ranges from 0 (no movement and no tonus adaption to a passive movement) to 20 (independent arm movement).

The *modified Rankin Scale (mRS) [28, 29]* and the *Barthel Index (BI)* [30-32] were assessed at day 2 after admission to hospital before the first treatment (baseline), at day 9 after treatment and at day 90 after stroke onset via a phone interview. The mRS is an ordinal scale for functional independence with reference to pre-stroke activities ranging from 0 (no symptoms) to 5 (severe disability), with a score of 6 allocated to patients who died. The BI is a validated measure of disability assessing 10 items of daily life and mobility activity with a total score ranging from 0-20 (lower scores indicating increased disability, 20 indicating all activities performed).

Motor power for inclusion criteria was measured using the *Medical Research Council Scale (MRCS)* [33], grading from grade 5 (normal power) to 0 (no contraction) differentiating between diminished power, movement against gravity, movement with gravity eliminated and flicker or trace contraction when attempting to move scoring 4,3,2 and 1, respectively.

4. Supplementary references

1. Epple, C., Maurer-Burkhard, B., Lichti, M.C., &Steiner, T. (2018). Vojta Therapy in Patients with Acute Stroke – A New Approach in Stroke Rehabilitation. *Phys Med Rehabil Int*. *5*(1): 1140-1146.
2. Vojta, V. (2004). *[Die zerebralen Bewegungsstörungen im Säuglingsalter – Frühdiagnose und Frühtherapie]*. (Vol. 8).Georg Thieme-Verlag
3. Ha, S.Y., &Sung, Y.H. (2016). Effects of Vojta method on trunk stability in healthy individuals. *J Exerc Rehabil*. *12*(6): 542-547. https://doi.org/10.12965/jer.1632804.402
4. Jung, M.W., Landenberger, M., Jung, T., Lindenthal, T., &Philippi, H. (2017). Vojta therapy and neurodevelopmental treatment in children with infantile postural asymmetry: a randomised controlled trial. *J Phys Ther Sci*. *29*(2): 301-306. https://doi.org/10.1589/jpts.29.301
5. Lim, H., &Kim, T. (2013). Effects of vojta therapy on gait of children with spastic diplegia. *J Phys Ther Sci*. *25*(12): 1605-1608. https://doi.org/10.1589/jpts.25.1605
6. Balzer, J., Marsico, P., Mitteregger, E., van der Linden, M.L., Mercer, T.H., &van Hedel, H.J.A. (2018). Influence of trunk control and lower extremity impairments on gait capacity in children with cerebral palsy. *Disabil Rehabil*. *40*(26): 3164-3170. https://doi.org/10.1080/09638288.2017.1380719
7. Titus, A.W., Hillier, S., Louw, Q.A., &Inglis-Jassiem, G. (2018). An analysis of trunk kinematics and gait parameters in people with stroke. *Afr J Disabil*. *7*: 310. https://doi.org/10.4102/ajod.v7i0.310
8. Van Criekinge, T., Truijen, S., Schroder, J., Maebe, Z., Blanckaert, K., van der Waal, C., et al. (2019). The effectiveness of trunk training on trunk control, sitting and standing balance and mobility post-stroke: a systematic review and meta-analysis. *Clin Rehabil*. *33*(6): 992-1002. https://doi.org/10.1177/0269215519830159
9. Witte, H., Hoffmann, H., Hackert, R., Schilling, C., Fischer, M.S., &Preuschoft, H. (2004). Biomimetic robotics should be based on functional morphology. *J Anat*. *204*(5): 331-342. https://doi.org/10.1111/j.0021-8782.2004.00297.x
10. Vojta, V. (1968). [Reflex creeping as an early rehabilitation programme]. *Z Kinderheilkd*. *104*(4): 319-330.
11. Vojta, V. (1970). [Reflex rotation as a pathway to human locomotion]. *Z Orthop Ihre Grenzgeb*. *108*(3): 446-452.
12. Vojta, V., &Peters, A. (2007). *[Das Vojta-Prinzip]*. (Vol. 3).Springer Heidelberg.
13. Murphy, T.H., &Corbett, D. (2009). Plasticity during stroke recovery: from synapse to behaviour. *Nat Rev Neurosci*. *10*(12): 861-872. https://doi.org/10.1038/nrn2735
14. Cramer, S.C., &Chopp, M. (2000). Recovery recapitulates ontogeny. *Trends Neurosci*. *23*(6): 265-271.
15. Pekna M, Pekny M, &Nilsson M. (2012). Modulation of neural plasticity as a basis for stroke rehabilitation. *Stroke*. *43*(10): 2819-2828.
16. Kwakkel, G., &Kollen, B.J. (2013). Predicting activities after stroke: what is clinically relevant? *Int J Stroke*. *8*(1): 25-32. https://doi.org/10.1111/j.1747-4949.2012.00967.x.
17. Collin, C., &Wade, D. (1990). Assessing motor impairment after stroke: a pilot reliability study. *J Neurol Neurosurg Psychiatry*. *53*(7): 576-579.
18. Duarte E, Marco E, Muniesa JM, Belmonte R, Diaz P, Tejero M, &Escalada F. (2002). Trunk control test as a functional predictor in stroke patients. *J Rehabil Med*. *34*(6): 267-272.
19. Franchignoni FP, Tesio L, Ricupero C, &Martino MT. (1997). Trunk control test as an early predictor of stroke rehabilitation outcome. *Stroke*. *28*(7): 1382-1385.
20. Sheikh, K., Smith, D.S., Meade, T.W., Brennan, P.J., &Ide, L. (1980). Assessment of motor function in studies of chronic disability. *Rheumatol Rehabil*. *19*(2): 83-90. https://doi.org/10.1093/rheumatology/19.2.83

21. Verheyden, G., Nieuwboer, A., Van de Winckel, A., &De Weerdt, W. (2007). Clinical tools to measure trunk performance after stroke: a systematic review of the literature. *Clin Rehabil*. *21*(5): 387-394. https://doi.org/10.1177/0269215507074055
22. Brott, T., Adams, H.P., Jr., Olinger, C.P., Marler, J.R., Barsan, W.G., Biller, J., et al. (1989). Measurements of acute cerebral infarction: a clinical examination scale. *Stroke*. *20*(7): 864-870.
23. Kasner, S.E. (2006). Clinical interpretation and use of stroke scales. *Lancet Neurol*. *5*(7): 603-612. https://doi.org/10.1016/S1474-4422(06)70495-1
24. Azouvi, P., Olivier, S., de Montety, G., Samuel, C., Louis-Dreyfus, A., &Tesio, L. (2003). Behavioral assessment of unilateral neglect: study of the psychometric properties of the Catherine Bergego Scale. *Arch Phys Med Rehabil*. *84*(1): 51-57. https://doi.org/10.1053/apmr.2003.50062
25. Azouvi, P., Samuel, C., Louis-Dreyfus, A., Bernati, T., Bartolomeo, P., Beis, J.M., et al. (2002). Sensitivity of clinical and behavioural tests of spatial neglect after right hemisphere stroke. *J Neurol Neurosurg Psychiatry*. *73*(2): 160-166.
26. Johansson, G.M., &Hager, C.K. (2012). Measurement properties of the Motor Evaluation Scale for Upper Extremity in Stroke patients (MESUPES). *Disabil Rehabil*. *34*(4): 288-294. https://doi.org/10.3109/09638288.2011.606343
27. Van de Winckel, A., Feys, H., van der Knaap, S., Messerli, R., Baronti, F., Lehmann, R., et al. (2006). Can quality of movement be measured? Rasch analysis and inter-rater reliability of the Motor Evaluation Scale for Upper Extremity in Stroke Patients (MESUPES). *Clin Rehabil*. *20*(10): 871-884. https://doi.org/10.1177/0269215506072181
28. Dromerick, A.W., Edwards, D.F., &Diringer, M.N. (2003). Sensitivity to changes in disability after stroke: a comparison of four scales useful in clinical trials. *J Rehabil Res Dev*. *40*(1): 1-8.
29. Rankin, J. (1957). Cerebral vascular accidents in patients over the age of 60. II. Prognosis. *Scott Med J*. *2*(5): 200-215. https://doi.org/10.1177/003693305700200504
30. Collin, C., Wade, D.T., Davies, S., &Horne, V. (1988). The Barthel ADL Index: a reliability study. *Int Disabil Stud*. *10*(2): 61-63.
31. Heuschmann, P.U., Kolominsky-Rabas, P.L., Nolte, C.H., Hunermund, G., Ruf, H.U., Laumeier, I., et al. (2005). [The reliability of the german version of the barthel-index and the development of a postal and telephone version for the application on stroke patients]. *Fortschr Neurol Psychiatr*. *73*(2): 74-82. https://doi.org/10.1055/s-2004-830172
32. Mahoney, F.I., &Barthel, D.W. (1965). Functional Evaluation: The Barthel Index. *Md State Med J*. *14*: 61-65.
33. Paternostro-Sluga, T., Grim-Stieger, M., Posch, M., Schuhfried, O., Vacariu, G., Mittermaier, C., et al. (2008). Reliability and validity of the Medical Research Council (MRC) scale and a modified scale for testing muscle strength in patients with radial palsy. *J Rehabil Med*. *40*(8): 665-671. https://doi.org/10.2340/16501977-0235
