# Supplementary material for: Vojta therapy improves postural control in very early stroke rehabilitation: a randomised controlled pilot trial
Source: Neurol Res Pract. 2020 Aug 20;2:23. doi: 10.1186/s42466-020-00070-4 (PMC7650119; doi:10.1186/s42466-020-00070-4)
Supplement: Supplementary file 5 — Additional file 4. Study Protocol Vojta Trial (2015). Original study protocol as published in Clinical Trials.gov and institutional homepage. [file 42466_2020_70_MOESM4_ESM.pdf]

Protocol
„Vojta Therapy in Early Stroke Rehabilitation-Trial"

(Translation of the german study protocol version 2 from Sept 03 2015)

Titel:
Improvement of Postural Control and Motor Function by Vojta Therapy in Early Stroke Rehabilitation of Stroke Patients - a Pilot Study and New Approach in Stroke Rehabilitation

Brief Title:
Vojta Therapy in Early Stroke Rehabilitation

German Title:
„Besserung der posturalen Steuerung in der Frühphase bei Patienten mit akutem ischämischem oder hämorrhagischem Schlaganfall durch Therapie nach dem Vojta-Prinzip im Vergleich zur Physiotherapie auf nicht-neurophysiologischer Grundlage („allgemeiner Physiotherapie zur motorischen Funktionsverbesserung") – die Höchster Vojta Stroke Studie"

NCT (ClinicalTrials.gov): 03035968

Study center:
Department of Neurology
Klinikum Frankfurt Höchst
Gotenstrasse 6-8
65929 Frankfurt am Main
Director: Prof. Dr. Thorsten Steiner, MME

Principal investigator (PI):
Corina Epple, MD
Department of Neurology
Klinikum Frankfurt Höchst
Gotenstrasse 6-8
65929 Frankfurt am Main
Phone:+49 (0)69-3106-2932
Email: corina.epple@klinikumfrankfurt.de

Principal physical therapist:
Barbara Maurer Burkhard
Department of Neurology
Klinikum Frankfurt Höchst
Gotenstrasse 6-8
65929 Frankfurt am Main
Phone:+49 (0)69-3106-2932

Board Status: Approved
Approval Number: FF 88/2015
Date of approval: October 14, 2015
Board Name: Ethics commission of the hessian regional medical board
Board Affiliation: Hessian Regional Medical Board
Phone: +4969 97672-0
Email: ethikkommission@laekh.de

1. Backround

In the last years there were revolutionary findings (i.e. thrombectomy) and an accumulation of a significant amount of evidence-based knowledge about acute stroke therapy and stroke prevention, but there is still a paucity of evidence-based knowledge about recovery and rehabilitation[1], although the evidence base for stroke rehabilitation has grown exponentially in the last 20 years.[2] Stroke is the major cause for permanent disability in adults. Unilateral motor weakness is one of the most common deficits resulting from stroke and one of the main causes of disability. It is still unclear, which physiotherapeutic approaches in stroke rehabilitation are most effective.[3] The Bobath-concept is one of the most widely used approaches in stroke rehabilitation within the western world, although several studies have failed to demonstrate superiority and showed partially even inferiority compared to other physiotherapy approaches.[4-6]

Modern approaches follow the idea, that functional improvement to a large extent relies on the use of compensatory movement strategies, enabling patients to learn to cope with their deficits. The Vojta therapy is based on a completely different approach: the reflex locomotion. Vojta described inborn movement sequences of reflex locomotion that are retrievable at all times. The therapist stimulates this innate patterns of movement by applying pressure to defined zones. There are ten zones available on the body, the arms and legs. The therapeutic use of reflex locomotion enables elementary patterns of movement in patients with impaired locomotor system, for example due to brain damage by stroke, to be restored once more, assuming that repeated stimulation of these “reflex-like” movements can lead to something like “new networking” within functionally blocked neuronal networks. After Vojta treatment, these patterns are more spontaneously available to the patient. Clinical experience shows, that Vojta therapy improves postural control, uprighting against gravity and goal-directed movements.

Initially Vojta therapy was applied mostly for newborns with central coordination disorders or spastic movement disoders. Although Vojta reflex locomotion is a sufficient therapeutic method for the promotion of normal posture control and the direct activation of respiratory muscles in children with cerebral palsy[7], its application in other diseases has not been well studied.[8] Vojta reflex locomotion has been reported to activate the trunk muscles and the deep muscles of the spine to regulate trunk stability and increase spinal rotation force, thereby enhancing postural control ability.[8] Nowadays it is also widely-used in adults, but concerning neurological diseases there is only some evidence for patients with multiple sclerosis[9] (indicating a positive effect of Vojta therapy) and for patients with paraplegia.[10,11] However to date no study has been performed for stroke.

Most studies on stroke recovery and neurorehabilitation have been conducted in so-called chronic patients, with stroke onset more than 6 months before, probably because this patients are easier to recruit and have a stable baseline, so that changes in performance can be

attributed to the experimental treatment.[12] Only few recovery trials have initiated restorative treatments within 7 days after onset and can therefore be classified as acute. There is an ongoing debate about the optimal timing of rehabilitation, although starting rehabilitation early is a widely accepted principle of care for people affected by stroke.[13] Delays to the initiation of rehabilitation seem to be associated with a poorer outcome and a longer length of stay in hospital for patients [14], furthermore motor training started around 5 days after stroke is more effective than training started at day 14 or day 30.[15]

The fact that there is a limited time window for the greatest motor recovery and increased receptivity to training regimens after injury is congruent with observations in animal models suggesting that there is about a month of heightened plasticity in the brain early after stroke when most recovery from impairment occurs [16,17]. For this reason in our trail we investigate acute stroke patients within 72 hours after stroke onset.

2. Purpose:

In our hospital we introduced Vojta therapy in 2013 on the stroke unit within the established treatment concept (predominantly Bobath concept). We and observed that Vojta therapy improved efficacy of other approaches, working with repetitive exercises by activation of automatic movement patterns and lead to an improvement of postural control, uprighting against gravity and goal-directed movements. Directly after Vojta therapy stroke patients showed an improvement of the posture and movement patterns, that showed definable consolidation tendency after a few days on the stroke unit. As an early effect we observed muslce fasciculation in the motoric target area -even in plegic limbs, appearance of movement sensation in neglect regions, as soon as vegetative (piloarective, vasomotoric, sudomotoric) reactions in the therapeutic area. Patients reported of sensoric effects as a heat sensation projected to the periphery, suggesting a systemic effect of Vojta therapy. This observations motivated as to further investigate Vojta therapy in stroke rehabilitaton.

We designed a randomized clinical trial (RCT) to compare Vojta therapy and conventional physiotherapy in patients with acute ischemic (AIS) or hemorrhagic stroke (ICH). This RCT will be the first trial to investigate improvement of postural control due to Vojta therapy in early rehabilitation of stroke patients, which is a very new approach in stroke-rehabilitation. The aim of the trial was to investigate Vojta therapy in acute stroke patients with severe hemiparesis patients within 72 hours after onset.

3. Hypothesis:

We hypothesise, that Vojta therapy improves postural control and motor function in early rehabilitation of stroke patients compared to conventional physiotherapy.

4. Study Design:

This is an investigator-initiated, prospective parallel-group, single-center, randomised controlled trial and will be conducted in the Department of Neurology at Frankfurt Höchst Hospital, Frankfurt, Germany. Patient recruitment and treatment will be performed by specifically trained personnel on a certified stroke unit (for mor details about certification please see http://www.dsg-info.de). All data will be monitored from clinical files as sources documents by the principal investigator.

5. Eligibility

5.1 Inclusion Criteria:

• Adult patients (> 18 years)
• CT or MRI proven acute ischemic (AIS) or hemorrhagic stroke (ICH) within 72h after onset of symptoms
• Severe hemiparesis (medical research council scale for muscle strength ≤2)
• premorbid modified Rankin Scale (mRS) ≤3
• maximum National Institute of Health Stroke Scale Score (NIHSS) 25
• Voluntary written consent by the patient

5.2. Exclusion Criteria:

• Severe cognitive impairment due to aphasia or dementia, prohibiting that physiotherapeutic challenges can be understood.
• Participation on another clinical trial
• Pregnancy

6. Screening and Randomisation:

All potentially eligible patients are screened on the first day after admission to the stroke unit and during the first 72h after stroke onset, in case of deterioration after admission. Eligible patient with written informed consent will be enrolled and randomly assigned (1:1) to receive usual stroke unit care with conventional physiotherapy (control group) or Vojta therapy (interventional group).

Arm	Therapy
interventional group	Vojta therapy
control group	conventional physiotherapy

Randomisation will be performed with sealed, opaque and sequentially numbered envelope. The investigators are instructed to assign the patient according to the envelope with the lowest randomisation number. Randomisation has to be done within 72 hours after onset of symptoms or after patients were last seen well and can be done by a physiotherapist or the investigator, who enrolles the subject. Stratification for stroke severity was disclaimed due to the small sample size.

7. Masking:

Masking of treatment is not possible because of the different appearance of the two physiotherapeutic approaches. Blinded assessment meanwhile hospital stay is not feasible, because it cannot be ensured that assessments are conducted by an evaluator physical therapist different from the treating physical therapist, due to logistic reasons. The trial is observer blinded only for the secondary clinical outcome Barthel Index and modified Ranking Scale at day 90. All other outcome assessors will have knowledge of the interventions assigned to individual participants.

8. Predefined Outcome Measures

8.1. Primary Outcome Measure:

Improvement of postural control measured with the trunc control test (TCT) on day 9 after admission to the hospital compared to baseline (Difference in scores on TCT scale between baseline and day 9)

8.2. Secondary Outcome Measures:

(i) Improvement of neglect measured with the Catherine Bergego Scale (CBS) part 5 and 6 on day 9 after admission to the hospital compared to baseline (Difference in scores on CBS scale between baseline before treatment and day 9 after treatment)

(ii) Improvement of arm motor function measured with the motor evaluation scale for upper extremity in stroke patients (MESUPES, part 1 to 4) on day 9 after admission to the hospital compared to baseline (Difference in scores on MESUPES between before treatment and day 9 after treatment).

(iii) Improvement of stroke severity measured with the National Institute of Health Stroke Scale (NIHSS) on day 9 after admission to the hospital compared to baseline (Difference in scores on NIHSS between baseline before treatment and day 9 after treatment).

(iv) Improvement of the Barthel Index (BI) on day 9 after admission to the hospital compared to baseline (Difference in scores on BI between baseline before treatment and day 9 after treatment)

(v) Improvement of the modified Rankin Scale (mRS) on day 90 after stroke compared to baseline (Difference in scores on mRS between baseline before treatment and day 90). The 90 day mRS is assessed via telephone interview by a blinded assessor.

(vi) Improvement of the Barthel Index (BI) on day 90 after stroke compared to baseline (Difference in scores on BI between baseline before treatment and day 90). The 90 day BI is assessed via telephone interview by a blinded assessor.

(vii) Improvement of the neglect (measured with the Catherine Bergego Scale) before and after every single intervention on day 2, day 5 and day 9.

(viii) Improvement of arm motor function (measured with the MESUPES) before and after every single intervention on day 2, day 5 and day 9.

The timing for the primary endpoint was chosen at day nine, because this is the mean residence time of stroke patients in the acute clinic before discharge to a rehabilitation center.

8.3 Safety Outcomes

All adverse events and serious adverse events are recorded and assessed by the investigators throughout the trial until day 90 according to standard definitions. All deaths until 3 months and the serious adverse during the hospital stay will evaluated and forwarded to a medical expert for assessment of relatedness to the study treatment.

9. Timeframes for Visits Outcome Measures

- Randomisation: Maximum 72h after stroke onset
- Test 1: on day 2 after admission to hospital (+/- 1 day)
- Test 2: on day 5 after admission to hospital (+/- 1 day)
- Test 3: on day 9 after admission to hospital (+/- 1 day)
- Test 4: on day 90 after stroke onset (+/- 5 days)

- The trunc control test (TCT) will be assessed at day 2 after admission to hospital before the first treatment (baseline), at day 5 and day 9 after treatment.
- The National Institutes of Health Stroke Scale (NIHSS) will be assessed at day 2 after admission to hospital before the first treatment (baseline) and at day 9 after treatment.
- The Catherine Bergego Scale (CBS) and the Motor Evaluation Scale for Upper Extremity in Stroke Patients (MESUPES) will be assessed at day 2, day 5 and day 9 after admission before and after treatment.
- The modified Rankin Scale (mRS) and the Barthel Index (BI) will be assessed at day 2 after admission to hospital before the first treatment (baseline), at day 9 after treatment and at day 90 after stroke onset via a phone interview.
- Investigators will record the occurrence of any serious adverse event and other safety outcomes that occur during hospital admission up to day 90.

Table 1: Outcome Measures

	Test 1 (day# 2 [+/-1]) (before therapy)	Test 2 (day# 5 [+/-1])	Test 3 (day# 9 [+/-1]) (after therapy)	Test 4 (day## 90[+/-5]) (phone contact)
TCT	x	x**	x	
NIHSS	x		x	
CBS	x*	x*	x*	
MESUPES	x*	x*	x*	
mRS	x		x	x
Barthel Index	x		x	x

Day #= day after admission to hospital
Day ## = day after stroke onset
X* = Assessment before and after therapy
X** = Assessment only after therapy
TCT = Trunc Control Test
NIHSS = National Institutes of Health Stroke Scale
CBS = Catherine Bergego Scale (item 5 and 6) (=Neglect Test)
MESUPES = motor evaluation scale for upper extremity in stroke patients (item 1 to 4 = MESUPES arm)
mRS = modified Rankin Scale

Table 2: trial schedule

Procedures	Screening visit	Baseline visit	Follow up visits			
	1) Screening day 1-3	2) Enrollement day 1-3	3) Test 1 day 2(+/-1)	4) Test 2 day 5 (+/-1)	5) Test 3 day 9 (+/-1)	6) Test 4 day 90 (+/-5)
Check inclusion/exclusion criteria	X	X				
Randomisation		X				
Informed consent		X				
Clinical examination	X	X	X	X	X	
Medical history	X					
Demographic data	X					
Concomitant medication		X			X	X
Adverse events		X	X	X	X	X
Imaging (CCT/MRI)	X					
NIHSS	X		X		X	
mRS	Premorbid-mRS		X		X	X
CBS (5+6) (neglect test)			X	X	X	
MESUPES arm (1-4)			X	X	X	
Trunc Control Test (TCT)			X	X	X	
Barthel Index			X		X	X

CCT=cranial computed tomography. MRI=magnetic resonance imaging. CBS=Catherine Bergego scale (item 5 and 6) (=Neglect Test). MESUPES= motor evaluation scale for upper extremity in stroke patients (item 1 to 4 = MESUPES arm).

Figure 1: time schedule

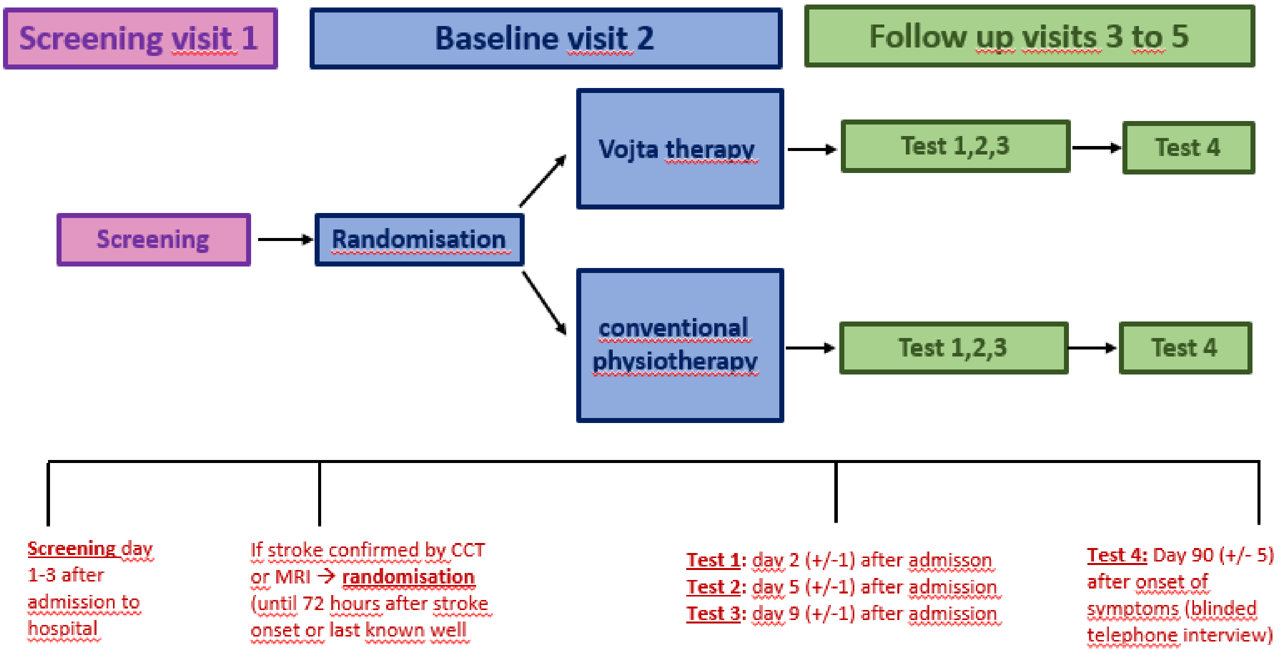

10. Outcome Assessments

10.1 Detailed description of assessment tools

***(i) Trunc Control Test (TCT)* [18-21]**

The TCT examines 4 items of axial movements:

1) rolling from a supine position to the weak side;
2) rolling from a supine position to the strong side;
3) sitting up from a lying-down position; and
4) sitting in a balanced position on the edge of the bed with feet off the ground for 30 seconds.

For each item can be scored 0, 12 or 25 points:

0= unable to perform movement without assistance;
12= able to perform movement, but in an abnormal style (Examples: pulls on bed clothes or monkey pole, or uses arms to steady self when sitting);
25= able to complete movement normally.

For the sitting balance item, a patient scores 12 if he needs to touch anything with his hands to stay upright, and 0 if he is unable to stay up (by any means) for 30 seconds. The TCT score is the sum of the scores obtained on the four tests and ranges from 0 (minimum) to 100 (maximum and indicating better performance). The examiner’s score must relate solely to the performance during the test.

***(ii) National Institutes of Health Stroke Scale (NIHSS)* [22-24]**

The NIHSS measures the severity of symptoms associated with cerebral infarcts and used as a quantitative measure of neurological deficit post stroke. The score was originally designed to assess differences among clinical trial interventions [24] and is compound out of 15 items assessing severity of impairment in different neurological systems (level of conciousness, speech, motor function, ataxia, sensory loss and others). Items are graded on a 3 or 4 point ordinal scale; a score of 0 typically indicates normal function in that specific ability. Score ranges from 0 to 42, higher scores indicating greater severity. Stroke severity may be stratified on the basis of NIHSS scores as mild (NIHSS 1-5), mild to moderately severe (NIHSS 5-14), severe (NIHSS 15-24) and very severe (NIHSS >25) [23].

***(iii) Catherine Bergego Scale (CBS) (=Neglect Test)* [25,26]**

The Catherine Bergego Scale is a standardised checklist to assess the presence and extent of neglect in patients with stroke and hemispatial neglect. The scale also provides a measure of neglect self-awareness (anosognosia). While the authors of this scale specify use of the CBS with patients with right hemispatial neglect, we will use this scale without modification for individuals with left hemispatial neglect. A functional scale consisting of 10 items related to neglect in everyday life (such as dressing, washing, eating, communicating, exploratory activities and moving around) is used. The CBS uses a 4-point rating scale to indicate the severity of neglect for each item, scoring 0 for no neglect, 1 for a mild neglect (patient always explores the right hemispace first and slowly or hesitantly explores the left side), 2 for a moderate neglect (patient demonstrates constant and clear left-sided omissions or collisions) and 3 for a severe neglect (patient is only able to explore the right hemispace). This results in a total score out of 30.

The therapist scores the patient on the following items:

1. Forgets to groom or shave the left part of his/her face
2. Experiences difficulty in adjusting his/her left sleeve or slipper
3. Forgets to eat food on the left side of his/her plate
4. Forgets to clean the left side of his/her mouth after eating
5. Experiences difficulty in looking towards the left
6. Forgets about a left part of his/her body (e.g. forgets to put his/her upper limb on the armrest, or his/her left foot on the wheelchair rest, or forgets to use his/her left harm when he/she needs to)
7. Has difficulty in paying attention to noise or people addressing him/her from the left
8. Collides with people or objects on the left side, such as doors or furniture (either while walking or driving a wheelchair)
9. Experiences difficulty in finding his/her way towards the left when traveling in familiar places or in the rehabilitation unit
10. Experiences difficulty finding his/her personal belongings in the room or bathroom when they are on the left side

The CBS takes approximately 30 minutes to administer. In order to save time and to spare our patients we abbreviated the CBS and assesed only items 5 and 6. This results in a total score of 6 (0 no neglect, 6 severe neglect).

(iv) Motor Evaluation Scale for Upper Extremity in Stroke Patients (MESUPES): [27,28]

The MESUPES is a objective evaluation scale designed to assess quality of movement of arm and hand function after stroke. The MESUPES was developed for use with adults with stroke. The scale consists of 17 items devided into two subscales: the MESUPES-Arm function with 8 items (scores 0-5) and the MESUPES-Hand function with 9 items (scores 0-2). With all items the test has a total score of 58 and a duration of 30 minutes. The MESUPES-Arm with 8 items has a total score of 40. The first four items are performed in supine; all other items are performed in a sitting position with hips and knees in 90° flexion and elbows on the table. The patient cannot be assessed if he cannot maintain an upright position for the tasks in sitting position. For this reason we decided to perform only items 1 to 4 in our trial, because the majority of patients with severe hemiparesis is not able to maintain an upright position in the early phase.

Items 1 to 4 start in a supine position, the head resting on a pillow, a small cylindrical pillow placed under the knees to support the legs, arms extended and resting on the table, forearms in pronation, fingers in a relaxed extended and adducted position. The patient is requested:

1) to put the hand on the stomach (score 0 to 5)
2) to put the hand back to the starting position (score 0 to 5)
3) to abduct the arm 0°-90° (arm extended, forearm in neutral position) (score 0 to 5)
4) to put arm back to the starting position (score 0 to 5).

The execution of these 4 items are scored in three consecutive phases:

A) *Passive score*: The task is performed passively and tone is evaluated (score 0 for hyper-or hypotonus and score 1 for an adequate adaption to the tone meanwhile the movement).

B) *Assisted score*: The therapist performs the movement while the patients assists and the presence of normal muscle contractions is scored (score 2)

C) *Independent score*: The patient performs the movement by himself and the range of motion that is executed in a qualitatively normal way is scored (score 3 if patient

performs part oft he movement; score 4 if patient performs the entire movement but slowly or with big effort; and score 5 if patient performs movement in normal speed).

The therapist should wait until tone is normalized before starting a new task. If the patient is not able to achieve a relaxed starting position, he is awarded a score of 0 for the item. A total score of 20 can be achieved with a range of 0 indicating no movement and no tonus adaption to a passive movement to 20 indicating an independing arm movement.

(v) Modfied Rankin Scale (mRS) [29,30] :

The modified Rankin Scale is a single-item global outcomes rating scale that categorises level of functional independence with reference to pre-stroke activities and is an ordinal scale ranging from 0 (*no symptoms*), 1 (*no significant disability despite symptoms; able to carry out all usual duties and activities*), 2 (*slight disability: unable to carry out all previous activities but able to look after own affairs without assistance*), 3 (*moderate disability: requiring some help, but able to walk without assistance*), 4 (*moderately severe disability: unable to walk without assistance, and unable to attend to own bodily needs without assistance*), to 5 (*severe disability: bedridden, incontinent, and requiring constant nursing care and attention*), with a score of 6 allocated to those who die. Assessment is carried out by asking the patient about their activities of daily living, including outdoor activities.

(vi) Barthel ADL Index (BI) [31-34] :

The Barthel scale or Barthel activities of dailiy life (ADL) index is a valid measure of disability. 10 items of daily life and mobility activity are assessed, including feeding, bathing, grooming, dressing, bowel control, bladder control, toileting, chair transfer, mobility and stair climbing. Items are rated based on the amount of assistance required to complete each activity. Each performance item is rated on this scale with a given number of points assigned to each level or ranking. A higher score is associated with a greater likelihood of being able to live at home with a degree of independence. There are several possible scoring techniques for the BI. The originally described index was scored in five-point increments, giving a score of 0-100. Collin et al. felt that this gave a misleading impression of its accuracy and so rescored it in one-point increments, giving a total score ranging from 0-20, with lower scores indicating increased disability. Each item is rated in terms of whether the patient can perform the task independently, with some assistance, or is dependent on help based on observation. In this trial we used the 20 point scale, because we had a validated german version for phone interview with a 20 point scale.

(vii) Medical Research Council Scale [35]:

Motor power for inclusion criteria was measured using the Medical Research Council scale, grading from power 5 (normal power) to 0 (no contraction) and differing between diminished power, movement against gravity, movement with gravity eliminated and flicker or trace contraction when attempting movement scoring 4,3,2 and 1, respectivley.

10.2: Summary for assessment tools:

- The TCT is a validated test to asses motor impairment and postural control after stroke. A range of 0 (patient is not able to turn around at all in lying position) to 100 (patient is able to sit for 30 seconds independently on the edge of the bed) points can be achieved.
- The NIHSS is used as a quantitative measure of neurological deficit in stroke patients. The 15 items score ranges from 0 to 42, higher scores indicating a more severe neurological deficit and a greater stroke severity.

- The CBS is a standardised checklist consisting of 10 items to assess the presence and extent of neglect in patients with stroke and hemispatial neglect, using a 4 point rating scale for each item (0 indicating no neglect and 3 indicating a severe neglect). In order to save time and to spare our patients we abbreviated the CBS and assessed only item 5 and 6. This results in a total score of 6 (0 no neglect, 6 severe neglect).
- The MESUPES is a clinical and research tool to qualitatively evaluate arm and hand function during recovery after stroke, comprising of 17 items pertaining to arm (8 items) and hand (9 items) performance. The whole tests takes up to 30 minutes, wherefore we performed in our trial items 1 to 4 of the MESUPES-arm. Furthermore only the first 4 items are performed in lying position, so that even severe effected patients were able to perfom the task. A total score of 20 can be achieved with a range of 0 (indication no movement and no tonus adaption to a passive movement) to 20 (indicating an independing arm movement).
- The mRS categorises level of functional independence with reference to pre-stroke activities and is an ordinal scale ranging from 0 (no symptoms) to 5 (severe disability), with a score of 6 allocated to patients who died.
- The BI is a validated measure of disability assessing 10 items of daily life and mobility activity with a total score ranging from 0-20 (lower scores indicating increased disability, 20 indicating all activities performed).

10.3 General comment:

The selection of the primary outcome parameter is a challenge in every clinical trial, but especially in rehabilitation trials, with a plenty of different assessment tools used in RCTs.[36] In our opinion postural control is an important prerequisite for further mobility which was the reason to choose the TCT as primary outcome parameter. The early control of sitting balance as tested with the TCT as a base for regaining standing balance and afterwards gait is an important factor for the final outcome at six-months.[16] We include the mRS and BI as a secondary outcome, although the European Stroke Organisation Outcomes Working Group[37] recommended the use of the mRS as a primary outcome measure in acute stroke trials. In the preperation of this trial we made a big effort to identify a probable assessment tool to objectify the hemispatial neglect, which is (together with aphasia, which was excluded) one of the most promiment focal cognitive deficits after stroke in clinical practice.[38] Due to feasibility in daily clinical routine and under consideration of the reduced resilience of acute stroke patients we choose the CBS as a standardized checklist to assess the presence and extent of neglect in patients with stroke and hemispatial neglect.[26] We selected assessment tools that are feasible in clinical routine and do not lead to an additional burden for the stroke patients.

11 Sample size and statistical analysis plan:

11.1 Sample size

This is a pilot trial. Vojta therapy has never been investigated in stroke patients. A sample size calculation for this pilot trial was not feasible, because desired parameters, as the estimated effect size or the standart deviation due to missing prior information or data in the literature could not be estimated. For feasability reasons we choose a sample size of 40 subjects (20 in each group) in order to complete recruitment in this monocentric trial within one to 1,5 years.

11.2 Statistical analysis plan

We plan an intention to treat analysis. For the primary endpoint (difference of scores in trunc control test between day 9 and baseline) an increase of 12 points (=12%) on the scale of the TCT was predefiened as a meaningful difference pragmatically, due to lack of data in the literature, because an improvement of 12 points in the TCT indicates a clinical relevant improvement of self-dependence (i.e. no more need of an auxiliary person, no more need for an adjuvant as edge of bed for moving). Normal distribution of the variables will be tested by the Shapiro-Wilk-test. For the primary endpoint we assume non-Gaussian distribution and we plan an analysis of variance with the non-parametric Mann–Whitney-U-test. The primary endpoint (difference of scores in trunc control test between day 9 and baseline) will be tested concerning the influencing factors a priori known to potentially affect outcome (age, sex, NIHSS > or <10 at baseline; stroke site, thrombolysis and thrombectomy) to test whether they affected the treatment. Complications occurring between baseline and day 9 will be analyzed with the exact Fisher Test. An interims analysis is not planned. We have not defined any discontinuation criteria. Statistical analysis will be made by the principal investigator with support of a statistical institute.

11.3 Data protection

Patient data will be pseudonymisised before statistically evaluation.

12. Therapeutical intervention and staff qualification

12.1 General comments concerning intervention and treatment time:

General management on the stroke unit of all included patients follows the standards of treatment defined in the contemporary European [39] (European Stroke Initiative [EUSI] and European Stroke Organization [ESO]) and national guidelines.

Patients will be either randomised to Vojta therapy or conventional physiotherapy within 72 hours after stroke onset. For ethical reasons, all patients received standard physiotherapy until randomisation in case randomisation is delayed e.g. by diagnostic procedures within and up to 72 hours. Elegible patients are preferred to get their MRI or CT scan as soon as possible in order to confirm their stroke (see „Imaging“).

Treatment sessions consist of 40 minutes once daily for 7 days for all patients, including treatment, mobilization and testing on weekend if necessary. Before testing on day 9 (+/-1 day) therefore all patients will have 7 treatment sessions. Assessments will be performed by two physiotherapist (one from the Vojta- one from the control group) following the four-eyes principle. In case of disagreement in scoring, a third physiotherapist should conduct an additional assessment.

12.2 Therapy for control group: Conventional physiotherapy

After randomisation the control group receives conventional physiotherapy. The physiotherapy programme for the control group includes repetitive sensomotoric exerscises of existing functions in the sense of a task-oriented training and movement facilitation of the restricted functions on the hemiparetic side. In addition, a balance training and gait training will be performed.

12.3 Therapy for interventional group: Vojta therapy

The interventional group will have Vojta therapy as physical therapy. Vojta therapy will be administered with stimulation of the breast zone, which is located between the 7th and 8th ribs, addition of other zones in order to support the activation was allowed. The starting position should be supine or lateral position with the head turned 30° in the direction of the stimulation, with the extremities lying naturally on the bed. No Vojta-therapist will treat patients allocated to the control group.

12.4 Qualification of physiotherapists and investigators

All physiotherapists involved in the trial must be licensed physiotherapists with a graduation (in germany vocational education for 3 years). Physiotheray students are not allowed to treat patients within this trial. All physiotherapists must have clinical experience for more than 2 years and have experience in treatment of stroke patients. Existence of an additional qualification as i.e. as bobath therapist is allowed for both groups. Therapists are requested not to treat following approaches of the bobath concept.

All physiotherapists treating the interventional group must have a certification (at least level A) of the international Vojta society as qualification (see http://www.vojta.com).

To minimise differences all staffs at the site were trained for both the intervention and assessments by the main investigator and the principal physical therapist. Investigators assessing the NIHSS and mRS should have a certification. For the other assessment tools no certification is required.

13. Imaging

Generally, imaging is performed only within the clinical indication as used in patients with ICH or AIS. Imaging usually includes a diagnostic CT-scan on admission and a follow-up imaging, generally a MRI and if not possible a CT-scan in order to confirm stroke, to determine extent of the infarction or in case of a ICH to review ICH volume and possible complications due to space occupying effects or determine ICH etiology. Patients are eligible only after a MRI or CT proven stroke. Therefore potential elegible patients are preferred to get their MRI or CT scan as soon as possible in order to confirm their stroke. No specific additional imaging is planned within the present trial.

14. General Definitions:

Baseline characteristics:

- Atrial fibrilation: no matter since when known
- Smokers: We defined a smoker as a current smoker or a participant, who had quit smoking in the last past 2 years.
- Handeness: as reported by patient (not assessed)
- Orthopaedic disease: as reported by patient or relatives, including prior surgery on spinal column or joints, prior traumas with disturbance of mobility, chronic lumbar pain syndrome and more.
- Dysphagie will be tested by the speech therapist (yes or no).

Acute stroke therapy:

- Treatment with recombinant tissue plasminogen activator and thrombectomy before randomisation are allowed.
- Surgical procedures (i.e. external ventricle drainage) before randomisation are allowed. Subjects must be able to sign the informed consent and to understand physiotherapeutic challanges.
- In case of deterioration with transfer to the intermediate care unit treatment will be continued there. If treatment or testing is not feasible (i.e. due to ventilation), testing should be performed one day later, if still within timeframe, otherwhise testing should cancelled. The final testing (visit 3) should then be performed as soon as the patient is testable again (if timeframe >day 10), even if he did not receive 7 treatment sessions in total.

15. Funding

This study is financially supported by a grant (8.600€) from the Hermann and Lilly Schilling foundation for medical research (Hermann und Lilly Schilling Stiftung für medizinische Forschung).

16. References

1. Donnan GA. Rehabilitation: the sleeping giant of stroke medicine. *Int J Stroke* 2013; **8**(1): 1.
2. Walker MF, Fisher RJ, Korner-Bitensky N, McCluskey A, Carey LM. From what we know to what we do: translating stroke rehabilitation research into practice. *Int J Stroke* 2013; **8**(1): 11-7.
3. Epple C, Maurer Burkhard B, Steiner T. Physiotherapy in acute stroke. [Physiotherapie bei akutem Schlaganfall] [article in german]. *DIVI* 2015; **7**: 70-7.
4. Kollen BJ, Lennon S, Lyons B, et al. The effectiveness of the Bobath concept in stroke rehabilitation: what is the evidence? *Stroke* 2009; **40**(4): e89-97.
5. Platz T. [Evidence-based arm rehabilitation--a systematic review of the literature]. *Nervenarzt* 2003; **74**(10): 841-9.
6. Van Peppen RP, Kwakkel G, Wood-Dauphinee S, Hendriks HJ, Van der Wees PJ, Dekker J. The impact of physical therapy on functional outcomes after stroke: what's the evidence? *Clin Rehabil* 2004; **18**(8): 833-62.
7. Lim H, Kim T. Effects of vojta therapy on gait of children with spastic diplegia. *J Phys Ther Sci* 2013; **25**(12): 1605-8.
8. Ha SY, Sung YH. Effects of Vojta method on trunk stability in healthy individuals. *J Exerc Rehabil* 2016; **12**(6): 542-7.
9. Laufens G, Poltz W, Buchstein G, Schmiegelt F, Stempski S. [Improvement in locomotion by combined treadmill/Vojta physiotherapy applied to selected MS patients] [Article in German]. *Phys Rehab Kur Med* 1999; **9**: 187-9.
10. Kellner L, Meiners T. [Soforteffekt in der Veränderung der Gehfähigkeit bei Patienten mit Querschnittlähmung nach der Vojta Therapie, ermittelt durch evidenzbasierte Gehtests]. *27 Jahrestagung der Deutschsprachigen Medizinischen Gesellschaft für Paraplegie e V DMGP*

Kloster Banz/Bad Staffelstein (DE) 1.–4. Juni 2014; **Abstract V 33**: http://www.dmgp-kongress.de/fileadmin/congress/media/dmgp/impressionen/DMGP2014_Abstractband.pdf. .
11. von Reumont AAbBPSiL. [Combination of treadmill and vojta physiotherapy after incomplete paraplegia] [German abstract] *Abstract presented at the German Physiotherapeutic Congress September 2012 in Leipzig, Germany* 2012 www.physio-deutschland.de/fileadmin/data/bund/events/BuKo2012/Abstracts/BuKo2012_Abstract_von_Reumont.pdf.
12. Krakauer JW, Carmichael ST, Corbett D, Wittenberg GF. Getting neurorehabilitation right: what can be learned from animal models? *Neurorehabil Neural Repair* 2012; **26**(8): 923-31.
13. Bernhardt J, Indredavik B, Langhorne P. When should rehabilitation begin after stroke? *Int J Stroke* 2013; **8**(1): 5-7.
14. Weinrich M, Good DC, Reding M, et al. Timing, intensity, and duration of rehabilitation for hip fracture and stroke: report of a workshop at the National Center for Medical Rehabilitation Research. *Neurorehabil Neural Repair* 2004; **18**(1): 12-28.
15. Biernaskie J, Chernenko G, Corbett D. Efficacy of rehabilitative experience declines with time after focal ischemic brain injury. *J Neurosci* 2004; **24**(5): 1245-54.
16. Kwakkel G, Kollen BJ. Predicting activities after stroke: what is clinically relevant? *Int J Stroke* 2013; **8**(1): 25-32.
17. Murphy TH, Corbett D. Plasticity during stroke recovery: from synapse to behaviour. *Nat Rev Neurosci* 2009; **10**(12): 861-72.
18. Collin C, Wade D. Assessing motor impairment after stroke: a pilot reliability study. *J Neurol Neurosurg Psychiatry* 1990; **53**(7): 576-9.
19. Duarte E, Marco E, Muniesa JM, et al. Trunk control test as a functional predictor in stroke patients. *J Rehabil Med* 2002 **34**(6): 267-72.
20. Franchignoni FP, Tesio L, Ricupero C, Martino MT. Trunk control test as an early predictor of stroke rehabilitation outcome. *Stroke* 1997; **28**(7): 1382-5.
21. Verheyden G, Nieuwboer A, Van de Winckel A, De Weerdt W. Clinical tools to measure trunk performance after stroke: a systematic review of the literature. *Clin Rehabil* 2007; **21**(5): 387-94.
22. Adams HP, Jr., Davis PH, Leira EC, et al. Baseline NIH Stroke Scale score strongly predicts outcome after stroke: A report of the Trial of Org 10172 in Acute Stroke Treatment (TOAST). *Neurology* 1999; **53**(1): 126-31.
23. Brott T, Adams HP, Jr., Olinger CP, et al. Measurements of acute cerebral infarction: a clinical examination scale. *Stroke* 1989; **20**(7): 864-70.
24. Kasner SE. Clinical interpretation and use of stroke scales. *Lancet Neurol* 2006; **5**(7): 603-12.
25. Azouvi P, Olivier S, de Montety G, Samuel C, Louis-Dreyfus A, Tesio L. Behavioral assessment of unilateral neglect: study of the psychometric properties of the Catherine Bergego Scale. *Arch Phys Med Rehabil* 2003; **84**(1): 51-7.
26. Azouvi P, Samuel C, Louis-Dreyfus A, et al. Sensitivity of clinical and behavioural tests of spatial neglect after right hemisphere stroke. *J Neurol Neurosurg Psychiatry* 2002; **73**(2): 160-6.
27. Johansson GM, Hager CK. Measurement properties of the Motor Evaluation Scale for Upper Extremity in Stroke patients (MESUPES). *Disabil Rehabil* 2012; **34**(4): 288-94.
28. Van de Winckel A, Feys H, van der Knaap S, et al. Can quality of movement be measured? Rasch analysis and inter-rater reliability of the Motor Evaluation Scale for Upper Extremity in Stroke Patients (MESUPES). *Clin Rehabil* 2006; **20**(10): 871-84.
29. Dromerick AW, Edwards DF, Diringer MN. Sensitivity to changes in disability after stroke: a comparison of four scales useful in clinical trials. *J Rehabil Res Dev* 2003; **40**(1): 1-8.

30. Rankin J. Cerebral vascular accidents in patients over the age of 60. II. Prognosis. *Scott Med J* 1957; **2**(5): 200-15.
31. Collin C, Wade DT, Davies S, Horne V. The Barthel ADL Index: a reliability study. *Int Disabil Stud* 1988; **10**(2): 61-3.
32. Heuschmann PU, Kolominsky-Rabas PL, Nolte CH, et al. [The reliability of the german version of the barthel-index and the development of a postal and telephone version for the application on stroke patients]. *Fortschr Neurol Psychiatr* 2005; **73**(2): 74-82.
33. Mahoney FI, Barthel DW. Functional Evaluation: The Barthel Index. *Md State Med J* 1965; **14**: 61-5.
34. Sulter G, Steen C, De Keyser J. Use of the Barthel index and modified Rankin scale in acute stroke trials. *Stroke* 1999; **30**(8): 1538-41.
35. Paternostro-Sluga T, Grim-Stieger M, Posch M, et al. Reliability and validity of the Medical Research Council (MRC) scale and a modified scale for testing muscle strength in patients with radial palsy. *J Rehabil Med* 2008; **40**(8): 665-71.
36. Ali M, English C, Bernhardt J, Sunnerhagen KS, Brady M, Collaboration VI-R. More outcomes than trials: a call for consistent data collection across stroke rehabilitation trials. *Int J Stroke* 2013; **8**(1): 18-24.
37. Lees KR, Bath PM, Schellinger PD, et al. Contemporary outcome measures in acute stroke research: choice of primary outcome measure. *Stroke* 2012; **43**(4): 1163-70.
38. Gottesman RF, Hillis AE. Predictors and assessment of cognitive dysfunction resulting from ischaemic stroke. *Lancet Neurol* 2010; **9**(9): 895-905.
39. European Stroke Initiative Writing C, Writing Committee for the EEC, Steiner T, et al. Recommendations for the management of intracranial haemorrhage - part I: spontaneous intracerebral haemorrhage. The European Stroke Initiative Writing Committee and the Writing Committee for the EUSI Executive Committee. *Cerebrovasc Dis* 2006; **22**(4): 294-316.
