# Supplementary material for: Vojta therapy improves postural control in very early stroke rehabilitation: a randomised controlled pilot trial
Source: Neurol Res Pract. 2020 Aug 20;2:23. doi: 10.1186/s42466-020-00070-4 (PMC7650119; doi:10.1186/s42466-020-00070-4)
Supplement: Supplementary file 7 — Additional file 7. Table of test results. Results of all tests for primary and secondary outcomes are provided in this table. [file 42466_2020_70_MOESM7_ESM.pdf]

Table: Results of Testing

Test(score [min-max]	Baseline *	Day 5 †	Day 9 ‡	Day 90
**Primary outcome**				
TCT (0-100)				
Vojta	25 (0-43)	49 (24-74)	72 (36.5-75)	
Control	56 (42.5-87)	50 (37-87)	74 (49-100)	
p-value	*0.006*	*0.316*	*0.581*	
Change TCT Day 9-baseline				
Vojta			25.5 (12.5-42.5)	
Control			0 (0-13)	
p-value			*0.001*	
**Secondary outcomes**				
MESUPES (0-20)				
Vojta	2·(0-5)	5.5 (2-7)	6 (4.5-12)	
Control	3 (1-10.5)	2 (0-12)	3 (1-15)	
p-value	*0.147*	*0.497*	*0.181*	
Change MESUPES Day 9-baseline				
Vojta			4 (1.5-6)	
Control			2 (0-5)	
p-value			*0.006*	
CBS (6-0)				
Vojta	4·(3-5.5)	3 (3-4)	3 (2-3)	
Control	3 (2-4)	2 (1-3)	2 (1-3)	
p-value	*0.022*	*0.013*	*0.191*	
Change CBS Day 9-baseline				
Vojta			2 (1-2)	
Control			1 (0-2)	
p-value			*0.054*	
NIHSS (0-42)				
Vojta	15 (12.5-16.5)		10.5 (9-12.5)	
Control	11.5 (10-14.5)		11 (8-12)	
p-value	*0.033*		*0.533*	
Change NIHSS Day 9-baseline				
Vojta			4 (2.5-5.5)	
Control			2 (0-4)	
p-value			*0.022*	
mRS (0-6)				
Vojta	5 (4-5)		4 (4-5)	4 (3-4.5)
Control	4 (4-5)		4 (4-4)	3.5 (3-4)
p-value	*0.098*		*0.16*	*0.361*
Change mRS			Day 9-baseline	Day 90-baseline
Vojta			0 (0-1)	1 (0-1)
Control			0 (0-1)	1 (0-1.5)
p-value			*0.987*	*0.943*
Barthel index (0-20)				
Vojta	3 (0-3.5)		5 (4-7)	8 (4-15)
Control	5 (1-9)		7 (2-11)	10 (5-17)
p-value	*0.078*		*0.735*	*0.522*
Change Barthel index			Day 9-baseline	Day 9-baseline
Vojta			3.5 (1.5-5)	6 (1-11)
Control			2 (0-4)	4.5 (2-9)
p-value			*0.083*	*0.796*

Data are median (interquartile range) or p-value. TCT = Trunk Control Test. MESUPES = motor evaluation scale for upper extremity in stroke patients (part 1 to 4 =MESUPES-arm). CBS = Catherine Bergego Scale (part 5 and 6). NIHSS = National Institutes of Health Stroke Scale. mRS = modified Rankin Scale. * Baseline testing before first treatment. ‡ Day 5 testing after treatment. † Day 9 testing after treatment.
