# Supplementary material for: Vojta therapy improves postural control in very early stroke rehabilitation: a randomised controlled pilot trial
Source: Neurol Res Pract. 2020 Aug 20;2:23. doi: 10.1186/s42466-020-00070-4 (PMC7650119; doi:10.1186/s42466-020-00070-4)
Supplement: Supplementary file 8 — Additional file 8. Adverse events during hospital stay. List of all adverse events during hospital stay (until discharge). [file 42466_2020_70_MOESM8_ESM.pdf]

Table: Adverse events during hospital stay

Adverse event (until discharge)	Interventional Group (n)	Control Group (n)
pneumonia by aspiration	1	3
pneumonia by congestion	1	0
urinary tract infection	4	1
pharyngitis	0	1
endocarditis	0	1
myocardial infarction	1	0
cerebral edema with coma	0	1
delirium	1	2
hypokalemia	1	0
hypernatremia	1	0
post stroke depression	2	2
hyperbilirubinemia	0	1
death	0	1
**Total**	**12**	**13**
